# Mapping Global Bushmeat Activities to Improve Zoonotic Spillover Surveillance by Using Geospatial Modeling

**DOI:** 10.3201/eid2904.221022

**Published:** 2023-04

**Authors:** Soushieta Jagadesh, Cheng Zhao, Ranya Mulchandani, Thomas P. Van Boeckel

**Affiliations:** Eidgenössische Technische Hochschule Zürich, Zurich, Switzerland (S. Jagadesh, C. Zhao, R. Mulchandani, T.P. Van Boeckel);; Sahlgrenska Academy, University of Gothenburg, Gothenburg, Sweden;; One Health Trust, Washington, DC, USA (T.P. Van Boeckel)

**Keywords:** zoonoses, viruses, hemorrhagic fever, Ebola, mpox, conservation, bushmeat, surveillance, pandemics, deforestation, wild meat, mammals, disease outbreaks

## Abstract

Human populations that hunt, butcher, and sell bushmeat (bushmeat activities) are at increased risk for zoonotic pathogen spillover. Despite associations with global epidemics of severe illnesses, such as Ebola and mpox, quantitative assessments of bushmeat activities are lacking. However, such assessments could help prioritize pandemic prevention and preparedness efforts. We used geospatial models that combined published data on bushmeat activities and ecologic and demographic drivers to map the distribution of bushmeat activities in rural regions globally. The resulting map had high predictive capacity for bushmeat activities (true skill statistic = 0.94). The model showed that mammal species richness and deforestation were principal drivers of the geographic distribution of bushmeat activities and that countries in West and Central Africa had the highest proportion of land area associated with bushmeat activities. These findings could help prioritize future surveillance of bushmeat activities and forecast emerging zoonoses at a global scale.

Bushmeat or wild meat refers to the meat of terrestrial wild mammals hunted primarily for human consumption in tropical and subtropical regions (*1*). Terrestrial wild mammals represent just 1.8% (≈0.003 gigatons of carbon [GtC]) of the global biomass of mammals (≈0.17 GtC) but are vastly outweighed by the biomass of domestic mammals raised for food (≈0.1 GtC) (*2*). However, >70% of zoonotic disease spillover events have been associated with wildlife and bushmeat (*3*,*4*). Hunting, preparing, and selling bushmeat (hereafter referred to as bushmeat activities) has been associated with high risk for zoonotic pathogen spillover due to contact with infectious materials from animals. Bushmeat activities provide opportunities for repeated pathogen transmission between animals and humans, leading to outbreaks, epidemics, and pandemics (*5*,*6*). For instance, Ebola virus spillover events and subsequent outbreaks in the Congo Basin have been traced back to hunters who were exposed to ape carcasses (*7*,*8*).

Bushmeat remains a staple source of protein among low-economic rural communities, where alternative proteins can be scarce (*9*,*10*). However, geographic distribution of bushmeat activities in rural areas remains insufficiently documented (*11*). The urban demand for bushmeat from rural areas is inconsistent and dependent on various reasons, including low cost compared with domestic meat, taste preferences, or social prestige (*12*). The hunted animal is often butchered and consumed immediately in rural areas (*13*). In regions where the urban demand is high, the animals are transported either live-caged or butchered and smoked to urban markets (*13*). Bushmeat activities pose a risk for zoonotic disease transmission regardless of setting (*14*), and the geographic and anthropologic heterogeneities in bushmeat activities renders surveillance for spillover risk challenging.

A recent study used the geographic range of endangered mammals to map mammal hunting for bushmeat and traditional medicine (*15*). Other mapping efforts, although accurate in capturing the market dynamics, have been restricted to local or regional settings (*16*,*17*). Research on bushmeat has been either biocentric, based on wildlife conservation (*18*), or anthropocentric, related to food security (*19*). Because zoonotic diseases known to be transmitted from wild mammals, such as mpox and Ebola, continue to emerge and expand geographically, an urgent need exists to integrate bushmeat activities into the epidemiology of emerging zoonoses. Efforts to document bushmeat activities have been sporadic and have not been synthesized geographically to enable objective prioritization and targeting of epidemiologic surveillance resources. However, to sustainably and effectively monitor at-risk areas for outbreak prevention and preparedness, bushmeat activity hotspots need to be identified on a global scale.

We mapped bushmeat activities in tropical and subtropical rural areas. We trained geospatial models that we calibrated by using published data and environmental and demographic covariates of bushmeat activities. We validated the capacity of the bushmeat activities map in predicting zoonotic disease emergence by using 2 established models of Ebola virus disease (EVD) (*20*,*21*). In addition, we identified 100 urban locations that could most benefit from increased surveillance for bushmeat activities.

## Methods

We used a multistep procedure to model the distribution of bushmeat activities. We modeled activities by using the following steps: collate datapoints from systematic literature search; prepare environmental and demographic covariates; fit model; conduct ensemble modeling; calculate the geographic area associated with bushmeat activities; and perform post hoc validation (Appendix).

### Data Collection

We searched for peer-reviewed reports on bushmeat hunting, handling, butchering, and selling by reviewing 3 electronic databases: Web of Science (https://www.webofscience.com), PubMed (https://pubmed.ncbi.nlm.nih.gov), and Google Scholar (https://scholar.google.com). We also searched websites for nongovernmental agencies, including International Union for Conservation of Nature (https://www.iucn.org), TRAFFIC (https://www.traffic.org), and the Center for International Forestry Research (CIFOR; https://www.cifor.org). We included studies with locations of bushmeat activities during January 1, 2000–February 1, 2022, and restricted the search to literature in English and French. 

**Figure 1:**
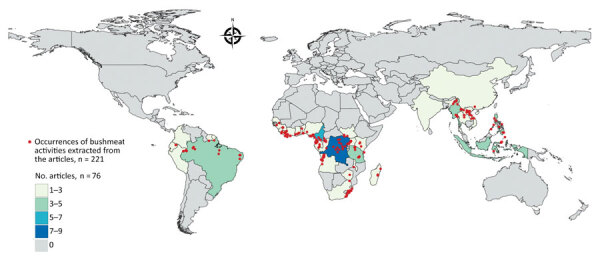
Geographic distribution of articles from the literature used to model a map of global bushmeat activities (hunting, preparing, and selling bushmeat) to improve zoonotic spillover surveillance. We extracted data from 76 articles. Red dots indicate occurrences of bushmeat activities (n = 221) in 38 countries, and colored shading indicated the number of articles extracted per country.

We identified 2,113 articles from all databases, of which 130 articles included geographic coordinates and precise locations of bushmeat activities. Among those 130 articles, we identified and included in the study 76 articles that were based in rural sites (defined as human settlements of <50,000 persons) (Figure 1). We excluded the other 54 articles because the locations included were urban sites (n = 28) or national parks without precise geographic coordinates of bushmeat activities (n = 26) (i.e., bushmeat was hunted or sold within the park). We excluded urban sites because different covariates could be associated with bushmeat activities between urban and rural sites, precise geographic coordinates were not given, and model prediction based on population density might be overestimated if a single pooled model was used for rural and urban sites (Appendix). 

We extracted 221 unique locations from the included studies and reports and georeferenced location latitude and longitude coordinates in decimal degrees. We used village or town centroids unless the exact location of markets were mentioned in the articles (Appendix). We created a search string and used PRISMA (https://www.prisma-statement.org) to create a flowchart of data extraction (Appendix Figure 1). 

### Environmental and Demographic Covariates

We extracted data from potential environmental and demographic covariates of bushmeat activities based on previous analyses (Appendix Table 1). Among those covariates, we developed 2 raster layers that we considered essential for predicting bushmeat prevalence. First, we developed a bushmeat species diversity raster from terrestrial mammal distribution data (*22*) and a list of mammals hunted and sold for commercial purposes for consumption, excluding mammals hunted as pests and trophies (*15*) (Appendix). We extracted a polygon layer of the distribution of 128 mammal species selected from the International Union for Conservation of Nature database of terrestrial wild mammals by using the species identification and then rasterized to 0.00833 degrees. Second, we constructed a raster of the distance to protected areas, such as natural parks, forest reserves, and wilderness areas (Appendix). We used data from World Geodetic System version 84 (GISGeography, https://gisgeography.com) to project all covariates and resampled by using a pixel resolution of 2.5 minutes of arc (0.04166 degrees), equating to ≈5 × 5 km resolution.

### Model Fitting and Evaluation

We selected 8 covariates with a recommended variance inflation factor (VIF) <10 (*23*) to account for potential collinearity among the covariates (Appendix Table 2). We used data on bushmeat activity extracted from the literature search datapoints, along with 1,000 randomly generated background points biased toward more populous areas as a proxy for reporting bias across the study area (*24*). We mapped bushmeat activities by using 4 models: MaxENT, random forest (RF), boosted regression tree (BRT), and Bayesian additive regression tree (BART). For each model, we used 80% of the datapoints (observed and background) for the training dataset; we used the remaining 20% of datapoints as the validation dataset (Appendix Figure 4). We fit and evaluated the base models by using area under the curve (AUC) and true skill statistic (maxTSS). 

We used 2 cross-validation (CV) methods and input covariates from R (The R Foundation for Statistical Computing, https://www.r-project.org) to prevent model overfitting: k-fold CV based on covariates from the SDMtune package (*25*) and environmental CV (EnvCV) with covariates from the blockCV package (*26*). We split the training data into 4-folds (k = 4) for both approaches. We only chose models with an AUC and maxTSS >0.5 after CV for hyperparameter tuning and to develop an ensemble model. The MaxENT model performed poorly (maxTSS = 0.47) in EnvCV, and we excluded it from further analysis. We also compared the models with a geographic null model to assess the predictive power of covariates (*27*).

### Model Optimization and Ensemble Modeling

We split data into training, validation, and testing sets for model optimization by tuning the appropriate hyperparameters for each model. We used the entire dataset in the optimized models to predict the global distribution of bushmeat activities. We stacked the model predictions from RF, BRT, and BART and used those as metacovariates for developing an ensemble model. We used a binomial logistic regression model in a hierarchical Bayesian framework with an intrinsic conditional autoregressive model (iCAR) (*28*) to assemble the model predictions. We validated the output ensemble prediction by using maxTSS and comparing deviance with a geographic null model. We generated the final 5 × 5 km resolution bushmeat activities raster from the mean probability from each pixel of the ensemble model. We took the SD of each pixel as an uncertainty metric. We used Pearson correlation between the mean probability and uncertainty raster to assess collinearity between the 2 metrics. To ensure that the prediction was focused in rural areas, we masked the urban centers by using an urban built-up area raster (*29*).

### Calculation of Area Associated with Bushmeat Activities

We reclassified the probability of bushmeat activities into 4 categories: very low probability (<0.2), low (0.2–0.5), intermediate (0.5–0.8), and high (>0.8). We then calculated the number of pixels per country in each category. For each country, we derived the proportion of area belonging to the high probability category by dividing the cumulative surface of those pixels by the area of the country.

### Post Hoc Validation

To evaluate the added value of the bushmeat activities raster map, we used it as a covariate in 2 established infectious disease risk mapping models and measured how the performance of these models improved. We chose 2 models of EVD (*20*,*21*), a zoonotic disease known to be transmitted through bushmeat. To reproduce the models, we used the dataset, predictors, and R code (if available) from the original published articles. To ensure the same number of predictor variables were used, we ran each model twice. We first used the MaxENT version 3.41 EVD model (*20*). We used a mask raster as the control in the first run of the MaxENT model, then compared its results with the bushmeat raster as a predictor covariate in the second run. We then used a BRT EVD model (*21*). For the first run, we used a randomly permuted bushmeat predictor as the control; for the second run, we used the extracted bushmeat covariate. We used a jackknife (leave-one-out) approach to determine the variable importance and AUC to compare the model performance without and with the bushmeat predictor layer (Appendix).

### Identifying Urban Locations for Future Bushmeat Activity Surveillance 

**Figure 2:**
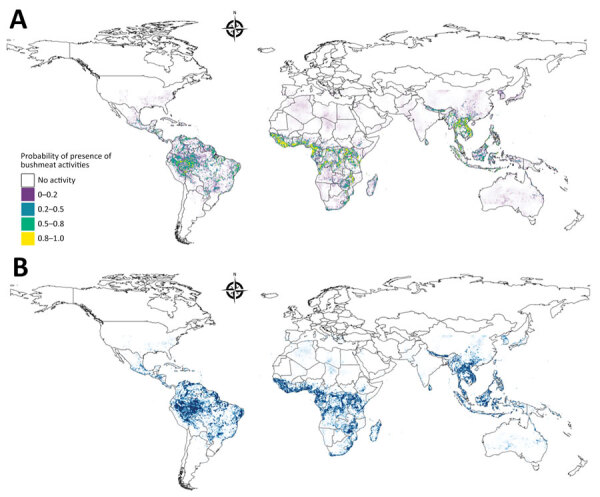
Model prediction and uncertainty maps for model of global bushmeat activities (hunting, preparing, and selling bushmeat) to improve zoonotic spillover surveillance. A) Distribution of bushmeat activities in the tropical and subtropical regions from an ensemble of 3 model predictions using a hierarchical binomial model with spatial autocorrelation. B) Map illustrating the uncertainty of predicted bushmeat activities represented by the SD of each pixel. Each pixel represents a 5 × 5 km area.

We identified 100 urban locations across the study area where we could conduct hypothetical surveys to maximize information gained from bushmeat activity surveillance. We quantified the necessity for additional surveillance (NS), a previously described measure (*30*), as the product of the uncertainty on bushmeat activity predictions and population density (Figure 2). We identified and placed a hypothetical survey on the pixel with the highest NS value, then gradually reduced NS around this first hypothetical survey by a 50-km radius (Appendix). We used the same procedure to add consecutive surveys by using the pixels with the highest NS until we identified 100 locations that could benefit from additional surveillance.

## Results

We conducted a systematic literature search and identified 2,113 studies reporting bushmeat activities (Appendix). To calibrate our model, we extracted 221 unique rural locations where bushmeat activities were reported from 76 articles (Figure 1). We extracted data on the taxonomic groups of bushmeat species from 59.2% (45/76) of the included articles. Even-toed ungulates (31%) were the most reported taxonomic group, followed by primates (28%), bats (15%), and rodents (15%) (Appendix Figure 3).

We modeled the geographic distribution of bushmeat activities by using the extracted occurrences and predictions of 3 geospatial models, RF, BRT, and BART (Figure 2). The resulting ensemble raster had a high maxTSS of 0.94 and was able to predict presence and absence of bushmeat activities. We identified an 859,765 km^2^ area, a superficial area ≈3.5 times the land area of the United Kingdom, as having a high probability (0.8–1) of bushmeat activities. Globally, the 3 countries with the largest proportion of their territory associated with bushmeat activities were Equatorial Guinea, Guinea-Bissau, and Liberia (Table 1). In Asia, Laos and Vietnam had the highest risk areas. The largest region, as classified by the United Nations geoscheme (https://www.un.org/geospatial), with bushmeat activities was in Central Africa (216,863 km^2^); the next highest was Southeast Asia (205,367 km^2^) (Appendix Table 18).

**Table 1:** Countries with high bushmeat activities in a study to map global bushmeat activities to improve zoonotic spillover surveillance by using geospatial modeling*

Country	Area with high probability for bushmeat activities, km^2^	Land surface area, km^2^	Percentage of country with high probability for bushmeat activities	Region
Equatorial Guinea	13,570	28,050	48.4	Central Africa
Guinea-Bissau	11,064	28,120	39.3	Central Africa
Liberia	28,955	96,320	30.1	West Africa
Malawi	25,498	94,280	27.0	East Africa
Sierra Leone	18,929	72,180	26.2	West Africa
Laos	49,354	230,800	21.4	Southeast Asia
Uganda	34,487	200,520	17.2	East Africa
Vietnam	48,230	310,070	15.6	Southeast Asia
Côte d’Ivoire	43,736	318,000	13.8	West Africa
Cameroon	56,355	472,710	11.9	West Africa

*High bushmeat activities (hunting, preparing, and selling bushmeat) are based on the proportion of high probability (>80%) areas in the ensemble raster.

Of the optimized RF, BRT, and BART models, the AUC and maxTSS were high and performed well against the geographic null model (average AUC 0.97 vs. 0.63; maxTSS 0.76 vs. 0.47) (Table 2). In both the RF and BRT models, the distribution of bushmeat activities was affected most by mammal richness, 42.2% in RF and 28.8% in BRT, and deforestation, 25.9% in RF and 17.2% in BRT. However, mean precipitation and mammal richness contributed most in the BART model (Appendix).

**Table 2:** Model predictive performance of a model map of global bushmeat activities to improve zoonotic spillover surveillance*

Model	AUC	maxTSS
Random forest	0.945	0.741
Boosted regression trees	0.945	0.758
Bayesian additive regression trees	0.952	0.775
Geographic null	0.633	0.472

*Predictive performance measured by AUC and maxTSS. Bushmeat activities are hunting, preparing, and selling bushmeat. AUC, area under the curve; maxTSS, maximum true skill statistic.

For the ensemble model, the hierarchical binomial model with iCAR performed better than the model without spatial autocorrelation and the geographic null model when we compared the deviance (Table 3). We calculated the global distribution of bushmeat activities from the mean value of the posterior distributions of probability per pixel of the ensemble model, and generated the uncertainty raster from the SD of the probability (Figure 2, panel A). We found no collinearity between the mean probability and the uncertainty per pixel (Appendix Figure 22).

**Table 3:** Comparison of model deviance and the percentage of deviance explained by the predictor covariates for model of global bushmeat activities to improve zoonotic spillover surveillance*

Model	Deviance	% Deviance explained	Covariates
Null	1153.835	0	None
Binomial	373.936	85	3 metacovariates†
Binomial iCAR	235.874	100	Addition of spatial autocorrelation

*Bushmeat activities are hunting, preparing, and selling bushmeat. iCAR, intrinsic conditional autoregressive. †Covariates included random forest, boosted regression tree, and Bayesian additive regression tree.

We conducted a post hoc validation by assessing the added value of the resulting map on the predictive performance of 2 established Ebola risk mapping models (*21*,*22*). Despite the negligible increase (<0.01) in AUCs of models with the bushmeat raster (Table 4), using bushmeat activities as a covariate contributed greatly to the distribution of EVD (Table 4; Appendix).

**Table 4:** Comparison performance for a map of global bushmeat activities to improve zoonotic spillover surveillance*

Model	Area under the curve		% Relative contribution of bushmeat activity
	Without bushmeat activity raster	With bushmeat activity raster	
EVD MaxENT	0.893	0.899	44.23
EVD BRT	0.880	0.887	17.06

*We compared area under the curve with and without bushmeat activities (hunting, preparing, and selling bushmeat) as predictor variable for EVD. BRT, boosted regression tree; EVD, Ebola virus disease.

**Figure 3:**
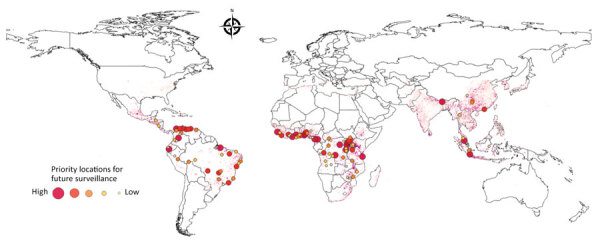
Predicted priority regions for future survey efforts in urban areas as determined by a model of global bushmeat activities (hunting, preparing, and selling bushmeat) to improve zoonotic spillover surveillance. The 100 priority locations identified are indicated by the necessity for surveillance, a previously described measure (*30*). Color and size of dots indicate high to low priority of needed surveillance efforts.

We used uncertainty levels on the map to identify 100 urban locations that could most benefit from future bushmeat surveillance efforts (Figure 3). The model predicted the largest number of surveys per country for Brazil (17 surveys) and the Democratic Republic of Congo (DRC; 15 surveys), the next highest was Colombia (8 surveys). South America (34 surveys) had the highest NS compared with South Asia (1 survey) and Central America (2 surveys) (Appendix Table 19). We provide model data in GitHub (https://github.com/soushie13/Bushmeat-related_activities) (Appendix).

## Discussion

We developed a global map of bushmeat activities in rural tropical and subtropical regions by using an ensemble geospatial modeling approach combined with 221 occurrence points extracted from previously published reports. The resulting map of 5 × 5 km pixels was consistent with published data on occurrence of local bushmeat activities (*16*,*17*), and with previous global mapping of efforts that focused on bushmeat hunting (*15*). We assessed the predictive capacity of our map by using 2 complementary approaches. First, we compared our model with a geographic null model, then we measured the improvement of existing risk mapping models for the occurrence of Ebola, after excluding our map in the model training process. Because we excluded urban areas from this study, we created an additional surveillance map to identify urban areas with the highest uncertainty of bushmeat activities and prioritized 100 urban locations for future surveillance.

Our results suggest that the largest areas associated with bushmeat activities were in Central Africa, Southeast Asia, and West Africa (Appendix Table 18). In most countries of Central Africa, the domestic livestock sector is negligible (Gabon, DRC, Congo) or limited (Cameroon, Central African Republic), leading bushmeat to be a crucial component of food security (*12*). Our results show that Equatorial Guinea in Central Africa had the highest proportion of land area associated with bushmeat activities. Equatorial Guinea is also home to the largest bushmeat market in Africa, Malabo Market on Bioko Island, where recent efforts to limit bushmeat sales through bans have been largely ineffective (*31*). Notable zoonotic diseases such as EVD and mpox have established origins from Central Africa in the 1970s and are believed to have been transmitted through bushmeat (*32*,*33*), demonstrating the significance of active surveillance of bushmeat activities in this region.

In Asia, Laos and Vietnam were the countries most associated with bushmeat activity (Table 1). A high volume of wildlife trade and established trade routes previously have been reported between Vietnam, Laos, and China (*34*,*35*). Studies have linked the origin of infectious reservoir sources of 2002–2004 SARS-CoV-1 outbreak that arrived at Guangdong markets and restaurants to Vietnam or Laos through a regional network (*36*,*37*).

Our study shows that data on bushmeat harvest in the Americas remain limited (10/76 studies included in data extraction), and only 10% of the predicted area was linked to bushmeat activities. Bushmeat commercialization was restricted to hidden markets in the Amazon Basin. Consumption in urban areas of the Americas has been unevenly studied (*12*) and is highly variable but not negligible, as previously thought because of large livestock production systems in South America (*38*,*39*). Our study also identified 34 urban sites in South America that would benefit from additional surveillance for bushmeat activities, highlighting that bushmeat activities remain underreported and understudied in that region (Figure 3).

As the risks of zoonotic spillover directly from wildlife are increasing, increased surveillance measures, including identifying and monitoring bushmeat hotspots, are urgently needed to predict spillover risk and enable early intervention (*5*,*40*). Virologic sampling and seroprevalence surveys that can be used to monitor spillover risk are costly and time consuming; thus, to optimize resources, those surveys require targeting locations where bushmeat is prevalent (*41*). Our approach to map the global distribution of bushmeat activities aims to help prioritize these efforts. Moreover, we validated this map for predicting the risk for EVD from previously established models (*20*,*21*) and found bushmeat activity was a major covariate in the distribution of EVD in Africa. Local governments and agencies could apply the necessity for additional surveillance map (Figure 3) to effectively monitor bushmeat activity sites that are often unreported, potentially unregulated, and previously unknown.

In this analysis, we used 8 environmental and demographic covariates to predict the geographic distribution of bushmeat activities. Mammal richness, deforestation, and precipitation had the greatest influence on the model distributions. Deforestation associated with development of logging roads enables easier access to the deeper forest and provides faster transportation of hunted meat to villages and towns (*42*). Control of deforestation and logging is urgently needed and could have far-reaching benefits for preventing bushmeat-associated zoonoses, as already established with EVD (*43*). In addition, studies show that precipitation effects bushmeat activities (*44*). In most areas, hunting pressure increases during the dry season when the water sources dry up, but in other areas, bushmeat hunting is preferred in periods of increased rainfall because the hunting sites become inaccessible to conservation patrols (*44*).

The first limitation of our study is that data on geographic sites of bushmeat hunting and selling are limited. Collecting reliable information on bushmeat-related activities is challenging because many species are protected under national laws, deterring informants from discussing their involvement to avoid incriminating themselves (*45*). Second, we did not independently collect data for this analysis, but that limitation is inherent to any modeling study attempting to map burden or risk by using passive surveillance data. Third, restriction of the spatial extent of the study area to the tropical and subtropical parts of the world might be considered an implicit bias; however, our intent was to focus on these regions as per the definition of bushmeat (*1*). Fourth, we did not quantify the distribution of zoonotic risk based on the taxonomic group of the mammal reservoir species as in other studies (*46*,*47*). However, the data we extracted from the literature search were insufficient to categorize the bushmeat by taxonomic groups because the species of bushmeat hunted was not consistently mentioned in the studies (*45*). Finally, we chose to exclude the urban sites for model calibration because they contained few locations (28 sites) with geographic coordinates of wet markets and chop shops, because different covariates may be associated with bushmeat activities between urban and rural sites, and because of overestimation of model prediction based on population density. However, we mitigated the exclusion of the urban sites by developing the necessity for additional surveillance map that detects urban areas that would benefit from future surveillance efforts (Figure 3; Appendix Table 19). A limitation of this map is that it is dependent on a single demographic variable, population density, and does not consider other factors, such as accessibility to the nearest city of population size.

Although geographic bushmeat data are limited, we attempted to characterize the distribution of bushmeat activities at a global scale to help identify priorities for action. Our study illustrates how environmental covariates, such as mammal richness, deforestation, and precipitation, affect bushmeat activities. Our findings highlight the increased need for conservation efforts, including prevention of habitat fragmentation and action against climate change. In addition to driving the bushmeat crisis, those factors also play a major role in the transmission of zoonoses (*48*). 

In conclusion, our findings contribute to the modeling and prediction of emerging zoonoses at global scale. The modeled findings can help target surveillance of bushmeat and bushmeat-related zoonotic spillovers by local reference laboratories established by the World Organization for Animal Health (*49*) and global outbreak prevention and preparedness initiatives like the Global Health Security Agenda (*50*). Our efforts to geographically synthesize bushmeat-related data could help prioritize future surveillance of bushmeat activities and forecast emerging zoonoses at a global scale.

Appendix Additional information on a modelling global bushmeat activities to improve zoonotic spillover surveillance using geospatial methods.
