# Supplementary material for: Mapping Global Bushmeat Activities to Improve Zoonotic Spillover Surveillance by Using Geospatial Modeling
Source: Emerg Infect Dis. 2023 Apr;29(4):742–50. doi: 10.3201/eid2904.221022 (PMC10045693; doi:10.3201/eid2904.221022)
Supplement: Appendix — Additional information on a modelling global bushmeat activities to improve zoonotic spillover surveillance using geospatial methods. [file 22-1022-Techapp-s1.pdf]

Article DOI: https://doi.org/10.3201/eid2904.221022

EID cannot ensure accessibility for supplementary materials supplied by authors. Readers who have difficulty accessing supplementary content should contact the authors for assistance.

Mapping Global Bushmeat Activities to Improve Zoonotic Spillover Surveillance by Using Geospatial Modeling

Appendix

All data needed to evaluate the conclusions in the paper are present in the Appendix and in the GitHub Repository (https://github.com/soushie13/Bushmeat-related_activities).

The distribution of bushmeat activities raster is available for download in its native 5×5 km resolution in the GitHub Repository along with the complied bushmeat activities coordinates and locations in need of additional surveillance. Please cite the paper when using the data.

Appendix Table 1. Environmental and demographic covariates and sources used in modeling the distribution of bushmeat activities

Covariate	Measures	Source
Minimum temperature	Minimum monthly temperature, °C	WorldClim, http://www.worldclim.org
Precipitation	Mean monthly precipitation, mm	WorldClim, http://www.worldclim.org
Deforestation	Aggregate of pixels with gross tree cover loss during 2000–2019	Earth Engine Partners, https://earthenginepartners.appspot.com/science-2013-global-forest/download_v1.7.html
Mammal richness	Number of mammal species per pixel	IUCN Red Book, https://doi.org/10.7927/H4N014G5
Bushmeat diversity	Number of mammal species hunted for bushmeat per pixel	This study; K.M. Marcoulides, et al., https://doi.org/10.1177/0013164418817803
Proximity to protected areas	Distance to protected areas	This study; January 2022 update of the WDPA and WD-OECM, https://www.protectedplanet.net/en/resources/january-2022-update-of-the-wdpa-and-wd-oecm
Accessibility to nearest city	Travel times to cities with population >50,000	A. Nelson, et al., https://doi.org/10.1038/s41597-019-0265-5
Population density	Estimated human population density, no. persons/km^2	IUCN, Gridded population of the world, version 4, https://doi.org/10.7927/H49C6VHW

Appendix Table 2. Glossary of terms and references used

Term	Definition	Key reference
Bushmeat or wild meat	Hunting of wildlife for human consumption in tropical areas. In this study, the scope of bushmeat activities was limited to the above definition, i.e., wildlife hunted primarily for human consumption in tropical and sub-tropical areas. Thus, trophy/game hunting, fur harvesting, hunting for traditional medicine, and hunting wildlife for leisure in temperate regions were not included in this study.	Milner-Gulland EJ, Bennett EL. Wild meat: the bigger picture. Trends Ecol Evol. 2003;18(7):351–7. https://doi.org/10.1016/S0169-5347(03)00123-X
Raster	A matrix of cells (or pixels) organized into rows and columns (or a grid) where each cell contains a value representing information, such as temperature.	ArcMap. What is raster data? https://desktop.arcgis.com/en/arcmap/latest/manage-data/raster-and-images/what-is-raster-data.htm
Spatial polygon	A set of spatially explicit shapes/polygons that represent a geographic location.	Michael T. Hallworth. Introduction to spatial polygons in R [cited 2022 Sep 15]. https://mhallwor.github.io/_pages/basics_SpatialPolygons
Variance inflation factor (VIF)	The VIF of an explanatory variable indicates the strength of the linear relationship between the variable and the remaining explanatory variables. A rough rule of thumb is that the VIFs greater than 10 give some cause for concern.	Forthofer RN, Lee ES, Hernandez M. (2007) '13 - Linear Regression', in R.N. Forthofer, E.S. Lee, and M. Hernandez (eds) Biostatistics (Second Edition). San Diego: Academic Press, pp. 349–386. https://doi.org/10.1016/B978-0-12-369492-8.50018-2.
Background points	A set of randomly generated spatial points to characterize the environment of the study region rather a guess to locate true absence locations.	Phillips SJ, Dudík M, Elith J, Graham CH, Lehmann A, Leathwick J, et al. Sample selection bias and presence-only distribution models: implications for background and pseudo-absence data. Ecol Appl. 2009;19(1):181–97. https://doi.org/10.1890/07-2153.1
Area Under the Curve (AUC)	A single scalar value that measures the overall performance of a binary classifier.	Hanley JA, McNeil BJ. The meaning and use of the area under a receiver operating characteristic (ROC) curve. Radiology. 1982 Apr;143(1):29–36. https://doi.org/10.1148/radiology.143.1.7063747
True Skill Statistic (maxTSS)	Based on the components of the standard confusion matrix representing matches and mismatches between observations and predictions.	Fielding AH, Bell, JF. A review of methods for the assessment of prediction errors in conservation presence/absence models. Environ Conserv. 1997;24:38–49. https://doi.org/10.1017/S0376892997000088
Ensemble modeling	A modeling ensemble is a group of models trained by different methods or algorithms, combined to produce a set of final predictions.	Elder J. Chapter 16, The Apparent Paradox of Complexity in Ensemble Modeling. In R. Nisbet, G. Miner, and K. Yale (eds), Handbook of Statistical Analysis and Data Mining Applications (Second Edition). Boston: Academic Press; 2018. pp. 705–718. https://doi.org/10.1016/B978-0-12-416632-5.00016-5
Hyperparameter	A parameter that is set before the learning process begins. These parameters are tunable and can directly affect how well a model trains.	DeepAI. Hyperparameter 2019 [cited 2022 Sep 18]. https://deepai.org/machine-learning-glossary-and-terms/hyperparameter
Prior	A probability calculated to express one's beliefs about this quantity before some evidence is taken into account.	DeepAI. Prior probability 2019 [cited 2022 Sep 23]. https://deepai.org/machine-learning-glossary-and-terms/prior-probability
Spatial autocorrelation	A special case of correlation, which is the global concept that two attribute variables X and Y have some average degree of alignment between the relative magnitudes of their respective values.	ScienceDirect. Comprehensive geographic information systems [cited 2022 Sep 18]. https://www.sciencedirect.com/referencework/9780128047934/comprehensive-geographic-information-systems

Data Collection: Literature Search and Data Extraction

Criteria for Considering Studies

Types of Studies

We considered studies with hunter, village, or offtake surveys and interviews on bushmeat hunting and/or consumption. Some studies' interviews focused on biodiversity loss due to bushmeat hunting. We also included studies analyzing the serology and/or SIV or similar viruses found in bushmeat markets or the vendors of bushmeat. We also included bushmeat market locations from trade reports.

Inclusion Criteria

Studies or reports with geographic coordinates or precise location of bushmeat, previously defined as meat of terrestrial wild mammals hunted primarily for human consumption in tropical and sub-tropical regions (*1*), hunting, selling, or preparation in the tropical and sub-tropical regions.

Exclusion Criteria

• Studies that did not include the precise location or name of the village or town bushmeat was sold or hunting took place.

• Studies with locations of urban centers with a population more than 50,000 and national parks and forest reserves.

Electronic Searches

Search Strategy for Identification of Studies

We attempted to identify all relevant studies regardless of publication status (published, unpublished, in press, ongoing), including preprints.

Three electronic databases were searched: Web of Science, PubMed (Medline Ovid host), and Google Scholar. All records were exported into the citation manager software Zotero and then uploaded to Rayyan QCRI for abstract review.

Search Terms

1) market* OR sale OR commercial OR hunt*,

2) AND: wildlife OR bushmeat OR “wild-life” OR “wild animal” OR “wild life”

A literature search via the Google Scholar was conducted with the addition of the search terms “TRAFFIC” / “CIFOR”/ “WWF.”

Search date: The literature search was conducted from the 6th December 2021 to the 4th February 2022.

Time period: 2000—present (February 2022), language: “English” and “French”

Supplementary searches: forward citation chasing on selected literature using citation chaser (https://estech.shinyapps.io/citationchaser).

The PRISMA flowchart with details of the flow of information through the different phases of the review is illustrated in Appendix Figure 1. It maps out the number of records identified, included, and excluded, and the reasons for exclusions. We later extracted geographic locations with or without coordinates for georeferencing. The locations extracted from the literature search were then georeferenced and projected using latitude and longitude coordinates and WGS84 datum. The study area extended from −110 west to 170 east and 40 north to −40 south.

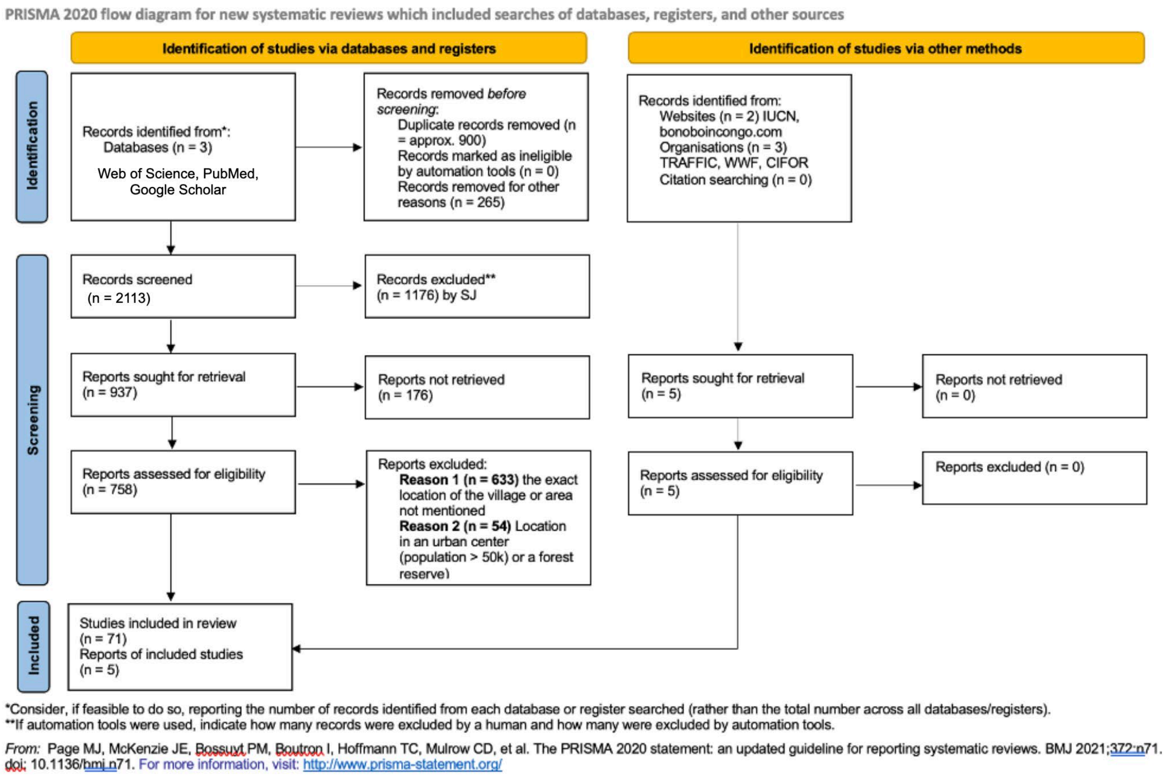

Appendix Figure 1. PRIMA flowchart detailing the research strategy.

The list of 76 studies and reports included for the study can be accessed at the GitHub repository under the document titled "included_studies.xslx." The spreadsheet includes the DOI, the authors, the year of publication, title of the article, and journal along with the name of the village or town, the longitudinal and latitudinal coordinates, the country, and the type of location, i.e., rural.

Data Extraction

The villages and towns with less than 50,000 population were extracted from the included 76 studies. The extracted data was then georeferenced and projected using latitude and longitude coordinates in a WGS84 datum. Out of the 224 occurrences, three were excluded due to faulty geo-localization. A final total of 221 unique occurrences were included in the modeling of the distribution of bushmeat-related activities. The georeferenced 221 occurrences can be accessed in its .csv format at the GitHub repository https://github.com/soushie13/Bushmeat-related_activities.

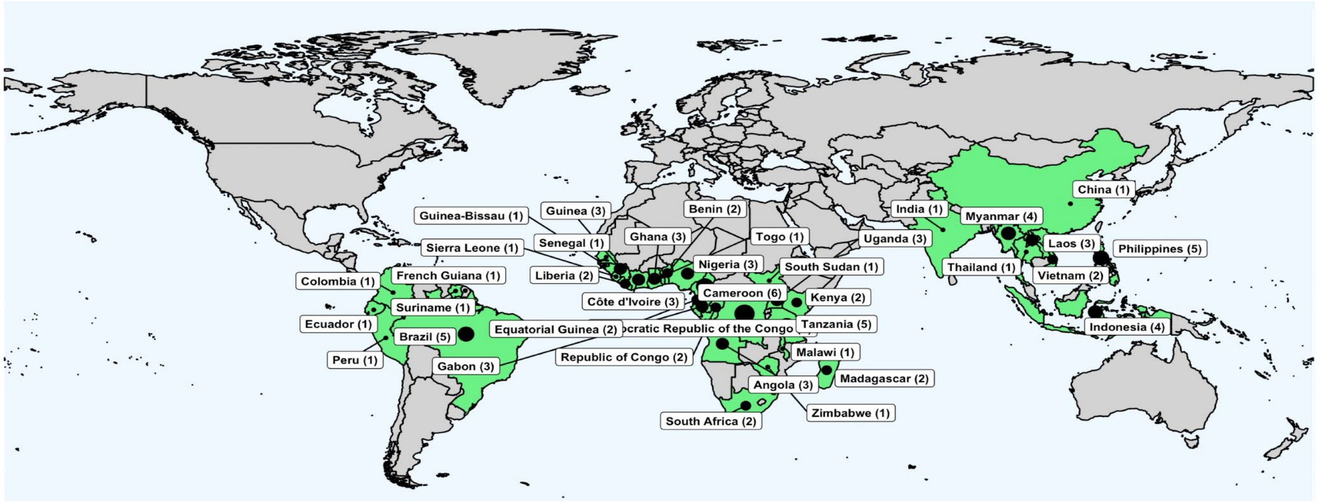

Appendix Figure 2. Countries (in green) from which articles were included following the systematic literature search.

Estimating the Comprehensiveness of the Search

- Search is not limited to the English language.
- Three bibliographic electronic databases were searched.
- Reports from organizations relevant to Bushmeat and wildlife conservation, such as TRAFFIC and WWF, were included in the search.
- Forward citation chasing the selected literature to ensure the comprehensiveness of the search.

Terrestrial Mammal Groups Reported in the Included Literature

We extracted mammal orders most hunted for bushmeat from 45 of the 76 included articles and reports.

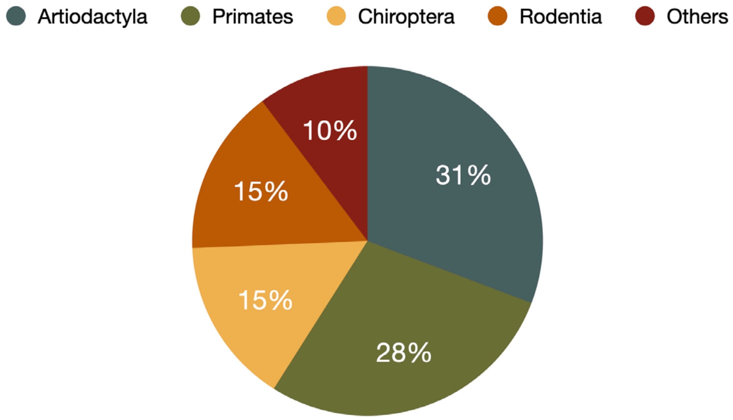

Appendix Figure 3. Distribution of taxonomic groups in the data extracted from systematic literature search.

Around 24 of 45 studies reported hunting of even-toed ungulates (Artiodactyla), followed by 22 studies reporting primates, 12 studies discussing bats (Chiroptera) and rodents (Rodentia). The other mammal orders reported included Canivora and pangolins (Phodolita).

Rationale behind Exclusion of Urban Sites

We chose to exclude urban sites due to the following reasons:1) different predictor covariates influencing bushmeat activities between urban and rural sites, 2) lack of precise geographic coordinates of urban sites and forest reserves, and 3) overestimation of model prediction based on population density

1. Differing Influencing Predictors between Rural and Urban Sites

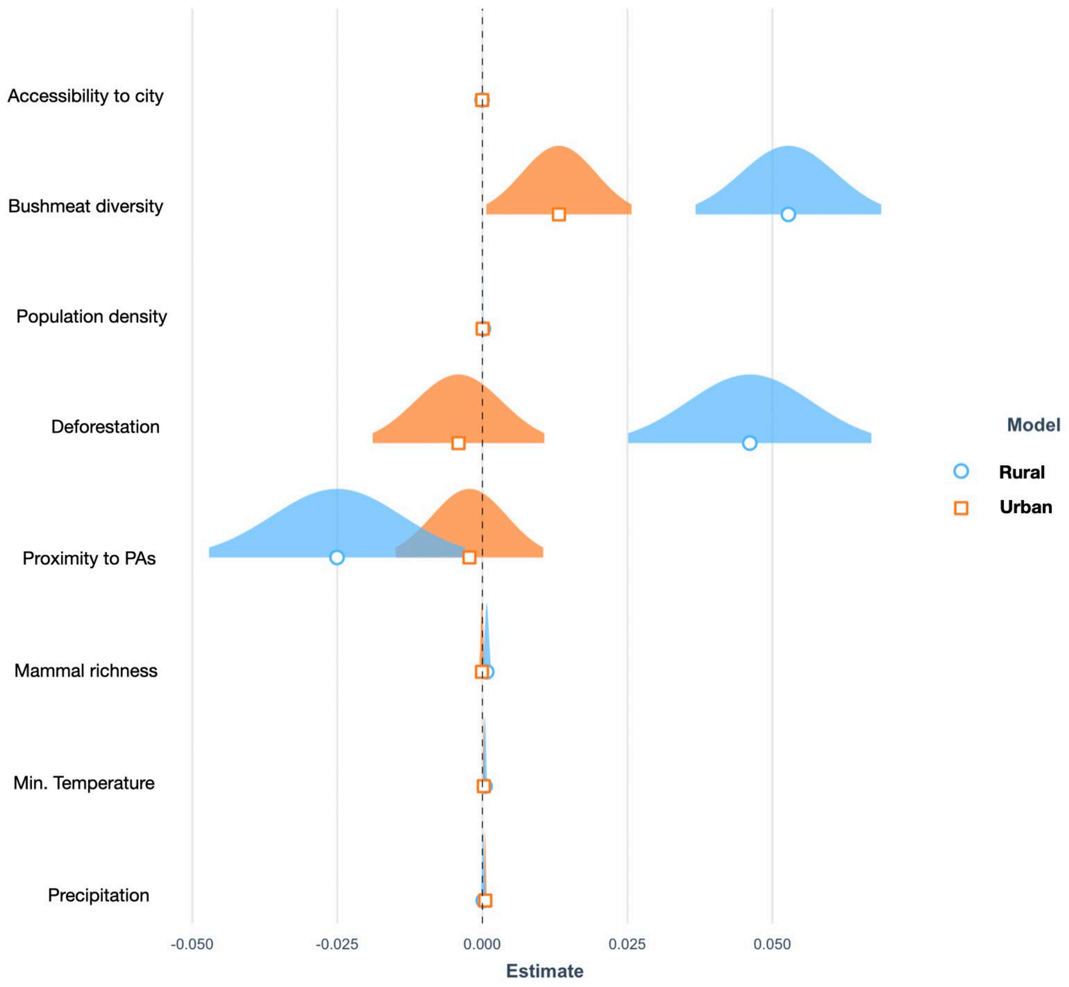

Appendix Figure 4. Comparison of factors influencing bushmeat activity in urban and rural areas.

On comparing the environmental and demographic predictors influencing the distribution of bushmeat activities between rural (n = 221) and urban areas (n = 76), we observed discrepancy in some of the factors. Deforestation, an established risk factor for bushmeat activities, had a significant positive impact in rural regions while having a negative effect in urban sites. As the number of accurately georeferenced urban sites were lower than rural sites and with the discrepancy in influencing factors, we chose to exclude the urban sites. The urban demand of bushmeat is dependent on distinct reasons including low cost in comparison to domestic meat, preference of taste, or social prestige. We were unable to find suitable anthropological covariates to address these factors influencing bushmeat activities in urban areas.

2. Lack of Precise Geographic Coordinates of Urban Sites and Forest Reserves

Coordinates of wet shops, chop shops, and restaurants where bushmeat is handled and sold were not available for over 60% (44/72) of the urban sites. Plotting centroids across large cities and towns would lead to inaccuracy in prediction, particularly in a fine resolution of 5×5 km. Similarly, we were unable to obtain precise coordinates of hunting of bushmeat in forest reserves.

3. Overestimation of the Model Prediction based on Population Density

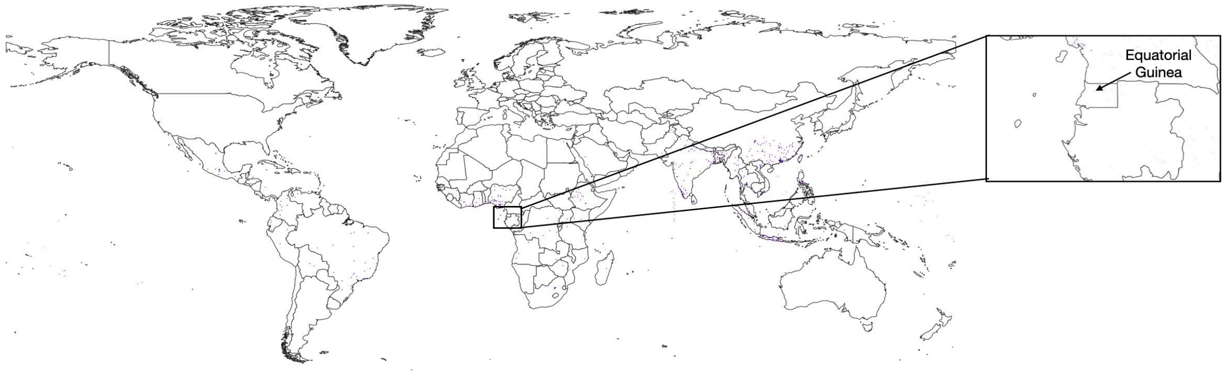

Appendix Figure 5. Overestimation of the model based on population density.

A 'RandomForest' model prediction using both combined rural and urban presence datapoints led to a poor prediction of bushmeat activities with missing predictions in key regions, such as Central Africa where bushmeat is widely consumed.

Study area

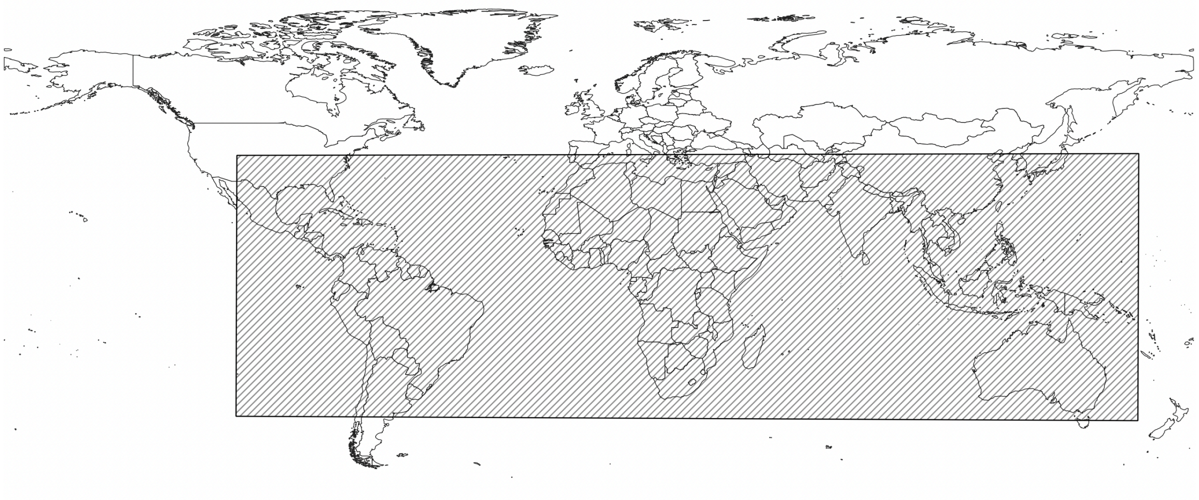

Appendix Figure 6. Geographic extent of the study area.

The study area was calculated using the minimum and maximum ranges from georeferenced datapoints extracted from the included 76 articles with a 15-degree extension on each side.

The extent of the study area in decimal degrees is as follows:

Maximum latitude (North): 27.194711

Minimum latitude (South): −32.459770

Maximum longitude (East): 126.837366

Minimum longitude (West): −76.624978

Environmental and Demographic Covariates List

We chose the predictor covariates based on known factors influencing bushmeat-related activities from previous studies and ecologic coherence (Appendix Table 3).

Appendix Table 3. List of predictor covariates

Covariate name	Measures	Source
Climate variables		WorldClim interpolated climate surfaces, http://www.worldclim.org
Minimum temperature, °C	Mean minimum temperature	
Maximum temperature, °C	Mean maximum temperature	
Precipitation	Mean monthly precipitation, mm	
Environmental variables		
Deforestation	Areas of gross tree cover loss	Global Forest Change 2000–2019 Version 1.7 from Hansen et al. (*2*), https://earthenginepartners.appspot.com/science-2013-global-forest/download_v1.7.html
Mammal richness	Number of mammal species	From Global Mammal Richness Grids, 2015 Release. International Union for Conservation of Nature – IUCN (*3*)
Bushmeat diversity	Number of mammals hunted for bushmeat	Dataset accessed from (*4*). Developed for this study. Description and process detailed below.
Proximity to protected areas	Distance to protected areas	Dataset accessed from https://www.protectedplanet.net/en/resources/january-2022-update-of-the-wdpa-and-wd-oecm. Developed for this study. Description and process detailed below.
Demographic variables		
Accessibility to the nearest city	Travel times to cities with population >50,000 in 2015	From Nelson et al. (*5*)
Population density	Population density estimates, no. persons/km^2	Data from Center for International Earth Science Information Network (*6*)
Gross domestic product	Gridded form for gross domestic product	From Gridded global datasets for Gross Domestic Product and Human Development Index over 1990–2015 (*7*)

Calculation of the Bushmeat Diversity Index

The list of mammal species hunted for meat were extracted from a previous publication, excluding those hunted as trophies, medicinal, or ornamental purposes (*8*). The list of mammals hunted for meat (Appendix Table 4) was filtered from the IUCN Terrestrial mammal polygons shapefile (accessed Jan 2022) using the identifier number (column name "Species_ID)" and binomial name ("Binomial") (Appendix Figure 7). Only the entry categories advised by the IUCN were included: Presence - 1 (extant); 2 (probably extant); and Origin – 1 (native); 2 (reintroduced). The entries under Presence- 6 (presence uncertain) and Origin- 5 (origin uncertain) were excluded. We selected 128 species from the IUCN terrestrial mammals list (Appendix Table 4).

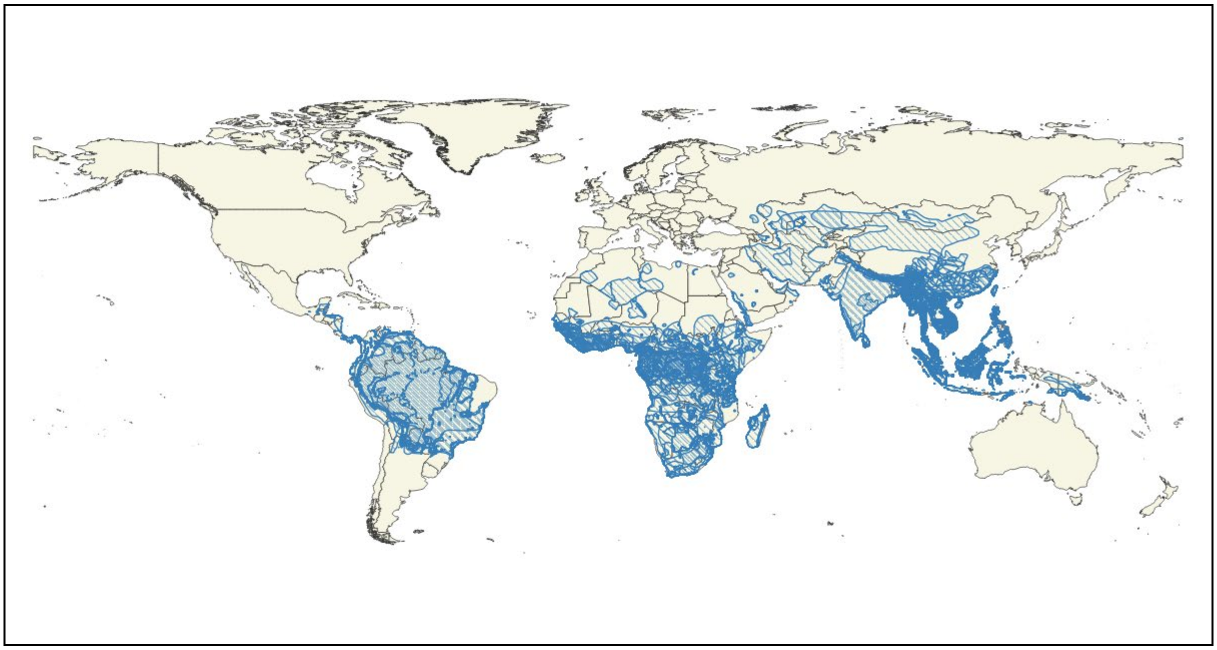

Appendix Figure 7. Terrestrial mammals "hunted for meat" polygons from the IUCN.

The filtered shapefile was further processed using QGIS version 3.16.14-Hannover. We used the "split vector layer" from the Data management tools under the Vector menu to split the polygons based on the species_ID. The individual polygon layers were rasterized to a resolution of 0.04166 degrees and extend to match the extent to the study area (−110 west to 170 east and 40 north to −40 south). The individual selected species rasters were summed using the raster calculator and the final Bushmeat diversity raster was created. The GeoTIFF file of this raster can be accessed from the GitHub repository.

Appendix Table 4. Binomial names of selected mammal species hunted for meat

Species	Species	Species	Species
Acerodon celebensis	*Lepus hainanus*	*Neofelis nebulosa*	*Rucervus duvaucelii*
Acerodon jubatus	*Lophocebus aterrimus*	*Nesolagus timminsi*	*Rucervus eldii*
Allochrocebus lhoesti	*Lutra sumatrana*	*Okapia johnstoni*	*Rusa alfredi*
Allochrocebus solatus	*Macaca arctoides*	*Oryx beisa*	*Rusa unicolor*
Arctocebus calabarensis	*Macaca leonina*	*Pan paniscus*	*Saiga tatarica*
Ateles chamek	*Macaca munzala*	*Pan troglodytes*	*Semnopithecus vetulus*
Babyrousa babyrussa	*Macaca nigrescens*	*Papio papio*	*Smutsia gigantea*
Boneia bidens	*Macaca pagensis*	*Pardofelis marmorata*	*Cercopithecus erythrotis*
Bos javanicus	*Macaca siberu*	*Pelea capreolus*	*Cercopithecus hamlyni*
Budorcas taxicolor	*Macaca tonkeana*	*Peroryctes broadbenti*	*Cercopithecus lomamiensis*
Bunolagus monticularis	*Macrogalidia musschenbroekii*	*Petaurus abidi*	*Cercopithecus lowei*
Chrotogale owstoni	*Mandrillus leucophaeus*	*Phataginus tetradactyla*	*Cercopithecus nictitans*
Colobus polykomos	*Manis crassicaudata*	*Phataginus tricuspis*	*Cercopithecus pogonias*
Colobus satanas	*Manis culionensis*	*Piliocolobus badius*	*Cercopithecus roloway*
Dendrohyrax validus	*Manis javanica*	*Piliocolobus lulindicus*	*Chiropotes satanas*
Desmalopex leucopterus	*Manis pentadactyla*	*Piliocolobus pennantii*	*Chiropotes utahickae*
Dorcopsis luctuosa	*Mazama bricenii*	*Piliocolobus preussi*	*Choeropsis liberiensis*
Eidolon dupreanum	*Mazama rufina*	*Piliocolobus semlikiensis*	*Sus ahoenobarbus*
Eidolon helvum	*Miopithecus ogouensis*	*Piliocolobus tephrosceles*	*Sus cebifrons*
Eulemur coronatus	*Muntiacus atherodes*	*Piliocolobus waldroni*	*Sus celebensis*
Eulemur macaco	*Cacajao calvus*	*Poiana leightoni*	*Sus oliveri*
Eupleres goudotii	*Capricornis rubidus*	*Pongo pygmaeus*	*Sus philippensis*

Species	Species	Species	Species
Felis nigripes	*Capricornis sumatraensis*	*Porcula salvania*	*Taeromys taerae*
Gazella subgutturosa	*Catagonus wagneri*	*Presbytis frontata*	*Tapirus indicus*
Genetta bourloni	*Cebus aequatorialis*	*Presbytis rubicunda*	*Tapirus terrestris*
Genetta piscivore	*Cercocebus chrysogaster*	*Propithecus coronatus*	*Tayassu pecari*
Gorilla beringei	*Cercocebus torquatus*	*Propithecus deckenii*	*Trachypithecus cristatus*
Gorilla gorilla	*Cercopithecus campbelli*	*Pteropus griseus*	*Trachypithecus francoisi*
Helarctos malayanus	*Cercopithecus diana*	*Pygathrix nemaeus*	*Tragelaphus derbianus*
Hippocamelus antisensis	*Cercopithecus dryas*	*Rhinolophus hillorum*	*Tragelaphus eurycerus*
Hoolock leuconedys	*Muntiacus vuquangensis*	*Rhinolophus maclaudi*	*Viverra megaspila*
Kobus megaceros	*Naemorhedus baileyi*	*Rhinopithecus strykeri*	*Zaglossus bartoni*

Development of the Proximity to Natural Protected Areas Raster

The polygon shapefiles from the World Database on Protected Areas (WDPA) were obtained (accessed Jan 2022). We extracted the WDPA of interest included the forest reserves, national parks, game reserves, and other terrestrial areas where bushmeat hunting is said to occur, i.e., IUCN management categories Ia (strict nature reserve), Ib (wilderness area), II (national park), and IV (habitat/species management area) were retained. Marine WDPA, urban parks, and other effective area-based conservation measures (OECMs) were excluded. Polygons under IUCN categories III (natural monument or feature), V (protected landscape/seascape), and VI (PA with sustainable use of natural resources) were also excluded.

A proximity raster (resolution of 0.04166 degrees) with a maximum radius of 50 km was generated. The GeoTIFF file of this raster can be accessed from the GitHub repository.

Model Flowchart

The modeling of the distribution of bushmeat activities was done through six steps: 1) datapoints collated from systematic literature search; 2) preparation of environmental and demographic covariates; 3) model fitting; 4) ensemble modeling; 5) calculation of the area associated with bushmeat activities; and 6) post-hoc validation (Appendix Figure 8).

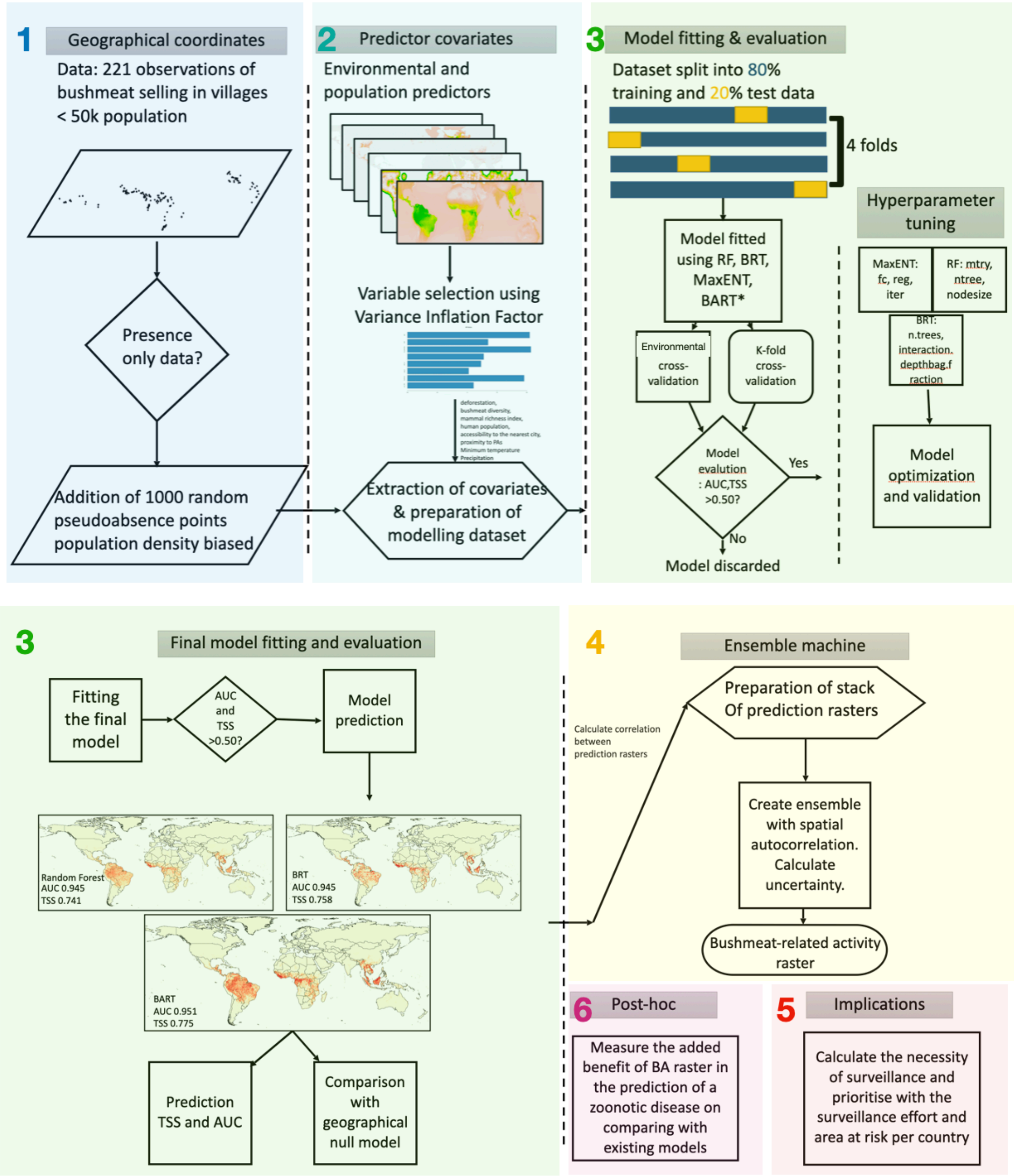

Appendix Figure 8. Flowchart of the model process.

Variance Inflation Factor

The variance inflation factor (VIF) was calculated for the ten covariates to avoid multicollinearity and a recommended threshold of 10 was established. The selected eight predictor covariates scored below threshold and were included in the models.

VIF 1 with 10 predictor variables.

VIF 2 with 9 predictor variables. GDP was removed to avoid correlation with population density. Population density is a well-established risk factor (*9*,*10*) for bushmeat-related activities and thus, was retained (Appendix Table 5).

Appendix Table 5. Predictor variables and variance inflation factors for VIF#2

Predictor variable	Variance inflation factor
Accessibility to city	1.110699
Bushmeat diversity	1.955751
Population density	1.030205
Deforestation	1.233203
Proximity to protected areas	1.277283
Mammal richness	2.073388
Minimum temperature	14.462742
Maximum temperature	12.079355
Mean precipitation	2.679160

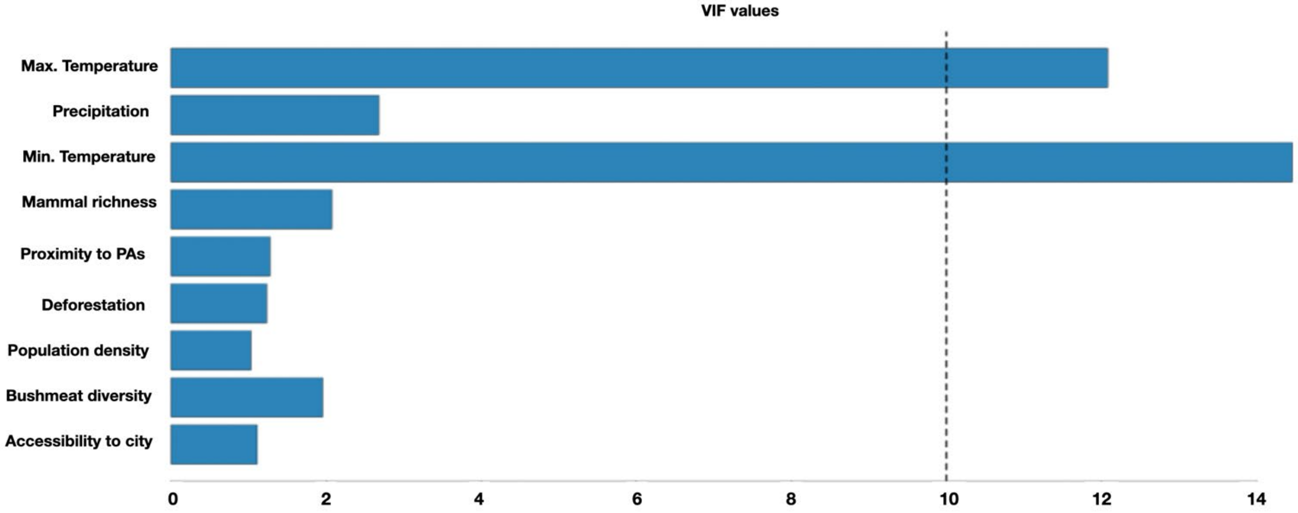

Appendix Figure 9. Variance Inflation Index with nine variables.

VIF 3 with 8 variables. Maximum temperature was excluded. The influence of climate change and freeze-free winters depend more on minimum temperature, which could therefore have more influence on zoonotic spillovers (Appendix Table 6).

Appendix Table 6. Predictor variables and variance inflation factors for VIF 3

Predictor variable	Variance inflation factor
Accessibility to city	1.107344
Bushmeat diversity	1.955222
Population density	1.026634
Deforestation	1.232670
Proximity to protected areas	1.276891
Mammal richness	2.069234
Minimum temperature	1.350941
Mean precipitation	2.048159

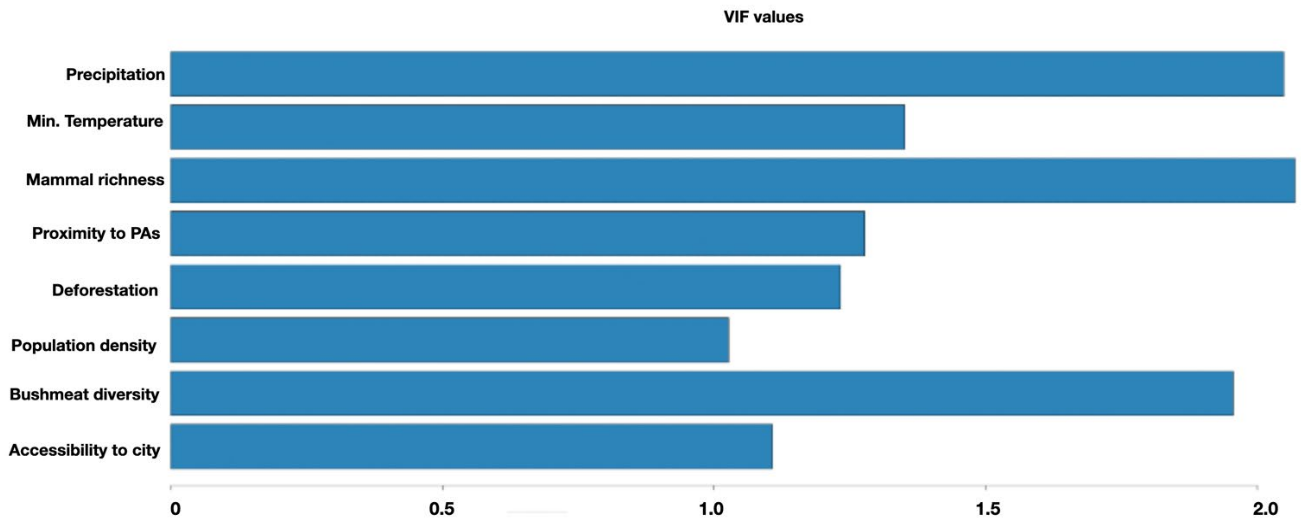

Appendix Figure 10. Variance inflation index with eight variables.

Cross-Validation Approaches

We used two cross-validation techniques, k-fold CV and environmental CV, to estimate the prediction ability of the model on unseen data and prevent overfitting of the model. The k-fold CV creates random clusters while environmental CV approach uses clustering methods to specify sets of similar environmental conditions based on the input covariates. Occurrence data corresponding to these clusters are assigned to a fold. Clustering of the predictor covariate data and occurrence data are done using kmeans.

In this study, we used 4-folds or clusters of the occurrence and background datapoints for both approaches. With 80% of the datapoints (observation and background) used as the training dataset, and the remaining 20% data attributed to the validation dataset. The eight predictor variables were used as covariates to define the environmental conditions for the environmental block CV.

Models with an AUC or maxTSS less than 0.5 following cross-validation were excluded (Appendix Figure 11).

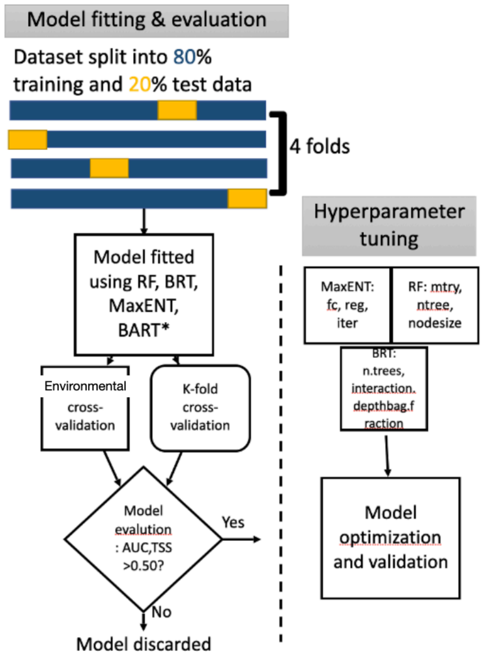

Appendix Figure 11. Cross validation approaches.

We used four modeling algorithms for the model fitting: MaxENT, Random Forest (RF), boosted regression tree (BRT), and Bayesian additive regression trees (BART). Only models with >0.5 AUC and maxTSS were selected. The models were also compared with a geographic null model to assess the predictive power of covariates. The geographic null model was generated by drawing a convex hull around the presence points.

Random Forest model

The 4 absence/background locations are NA for some environmental variables and were thus excluded, resulting in 996 background points that are biased based on population density. We used R packages 'randomForest' (*11*) and dismo (*12*) to fit Random Forest (RF) models (*13*) (Appendix Table 7).

Appendix Table 7. Predictor variables and variance inflation factors

Metric	CV random forest	envCV random forest	Final random forest
Area under the curve	0.9494543	0.8399409	0.9454089
True skill statistic	0.7669548	0.6041012	0.7408634

*CV, cross-validation; envCV, environmental cross-validation.

For model optimization using hyperparameter tuning, we used Random search using different combinations of tunable parameters. For RF, the tunable parameters (hyperameters) are:

mtry (number of variables randomly sampled as candidates at each split), ntrees (number of trees to grow), and node size. The parameters were later confirmed using individual grid searches.

Random Search: Node Size and ntrees

We tested the combination of node sizes (1–10) and ntrees up to 500 and evaluated the model performance using (Appendix Table 8, Appendix Figure 12).

.

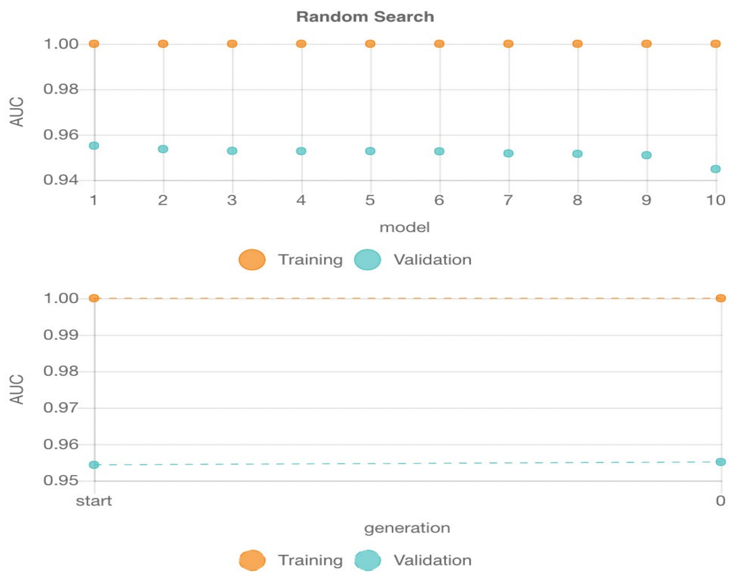

Appendix Figure 12. Random search approach for model evaluation of RF model using AUC.

Appendix Table 8. Area under the curve test of random forest model

mtry	ntree	Nodesize	Test area under the curve
2	192	1	0.9550594
2	315	8	0.9536318
2	340	5	0.9528323
2	453	5	0.9527181
2	58	4	0.9526610
2	299	4	0.9526039
2	400	7	0.9517474
2	303	7	0.9514619
2	332	2	0.9509479
2	39	2	0.9447807

We also evaluated the model performance using TSS (Appendix Table 9, Appendix Figures 13, 14).

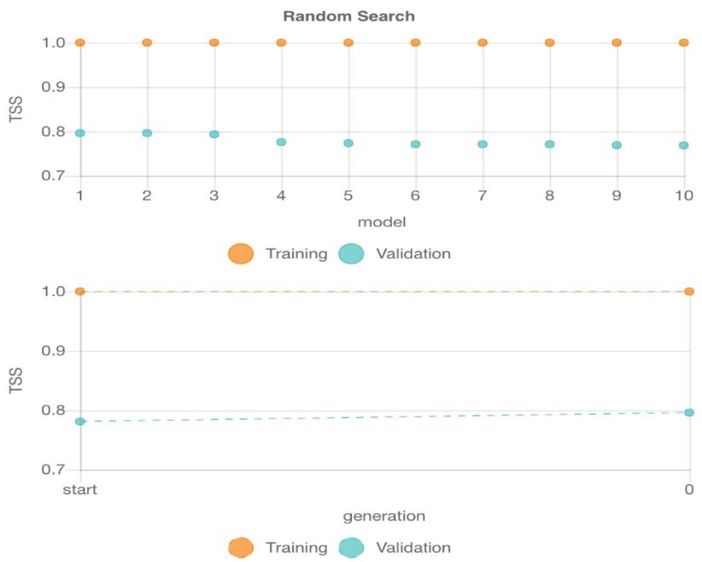

Appendix Figure 13. Random search approach for model evaluation of RF model using TSS.

Appendix Table 9. True skill statistic test of random forest model

mtry	ntree	Nodesize	Test true skill statistic
2	315	8	0.7961398
2	340	5	0.7961398
2	192	1	0.7935130
2	453	5	0.7760393
2	332	2	0.7734125
2	400	7	0.7710142
2	299	4	0.7710142
2	58	4	0.7710142
2	303	7	0.7688442
2	39	2	0.7686158

Grid search: ntrees

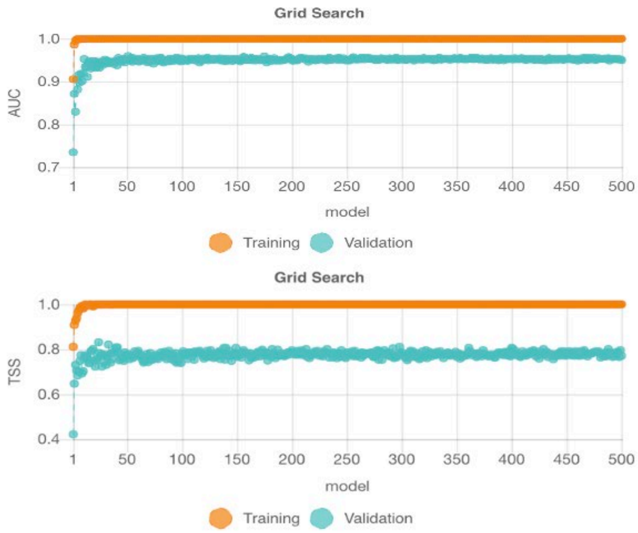

Appendix Figure 14. Hyperparameter tuning of RF model using a grid search approach for number of trees parameter.

Above figures (Appendix Figures 13, 14) evaluate the model performance using AUC and TSS for a single hyperparameter.

RF Final Model Parameters

ntree = 500, nodesize = 5, mtry = 2

Appendix Table 10. Random forest variable model permutation importance

Variable	Permutation importance	SD
Mammal richness	42.2	0.003
Deforestation	25.9	0.002
Bushmeat diversity	8.3	0.002
Proximity to protected areas	7.6	0.001
Minimum temperature	6.8	0.001
Accessibility to city	5.0	0.000
Annual precipitation	2.3	0.000
Population density	2.0	0.000

RF Jackknife Test

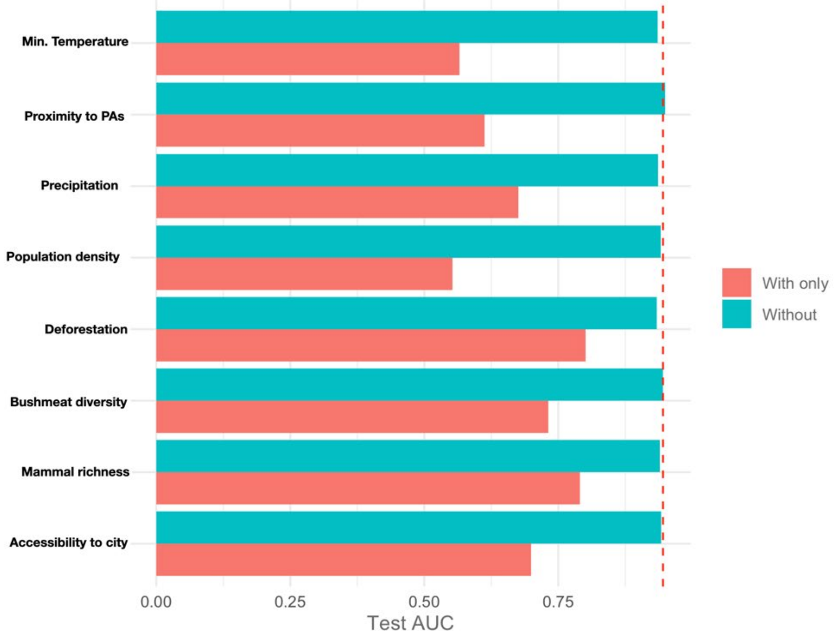

Appendix Figure 15. Jackknife test to test the variable contribution for the RF model, permutation set to 10.

RF ROC

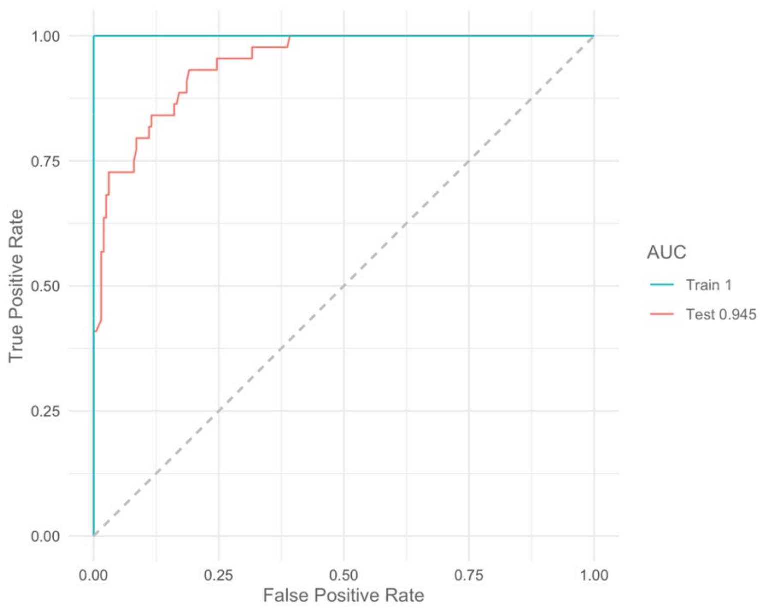

Appendix Figure 16. ROC curves of the RF model.

RF Model Prediction

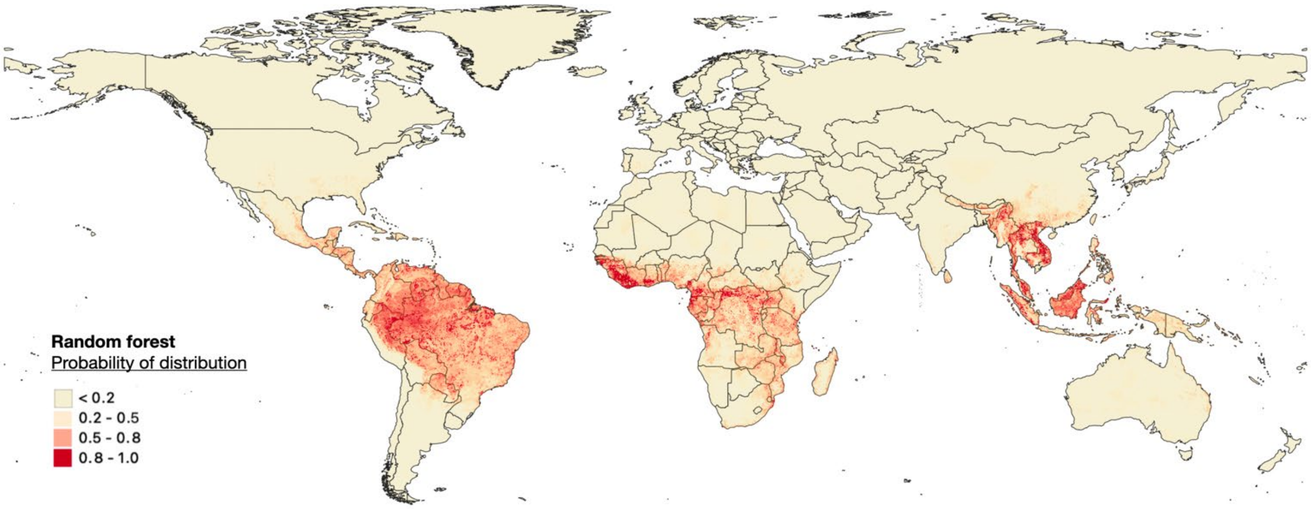

Appendix Figure 17. RF model prediction of the distribution of bushmeat activities.

Boosted Regression Trees

We used R packages ‘gbm’ (*14*) and ‘dismo’ (*12*) to fit BRT models (*15*) (Appendix Table 11).

Appendix Table 11. Boosted regression tree model fit

Metric	CV	evnCV BRT	Final BRT
Area under the curve	0.9186463	0.8318459	0.9452947
True skill statistic	0.7281369	0.6149154	0.7583371

*BRT, boosted regression tree; CV, cross-validation; envCV, environmental cross-validation.

For BRT, the tunable parameters are: distribution, n.trees (maximum number of grown trees), interaction depth, shrinkage, bag fraction. We set the distribution to “bernolli,” bag fraction = 0.5, shrinkage = 1, and interaction depth = 1. We used grid search to tune a single parameter, n.trees (Appendix Figure 18).

Grid search: n.trees

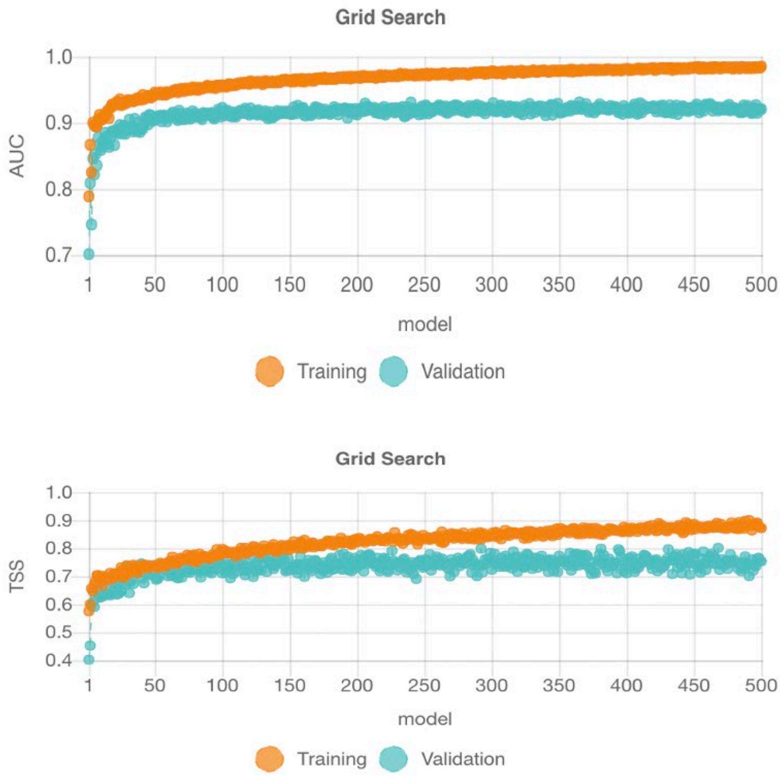

Appendix Figure 18. Hyperparameter tuning of BRT model using a grid search approach for number of trees parameter.

Appendix Figure 18 evaluates the model performance using AUC and TSS for a single hyperparameter.

Final BRT model parameters: n.trees = 500, learning.rate = 0.0025, bag.fraction = 0.5

BRT: Variable Importance

Appendix Table 12. Variable importance in boosted regression tree model

Variable	Permutation importance	SD
Mammal richness	28.8	0.007
Deforestation	17.2	0.006
Minimum temperature	13.4	0.004
Accessibility to city	12.5	0.004
Population density	12.0	0.003
Proximity to protected areas	7.5	0.003
Annual precipitation	7.0	0.001
Bushmeat diversity	4.5	0.002

BRT Jackknife test

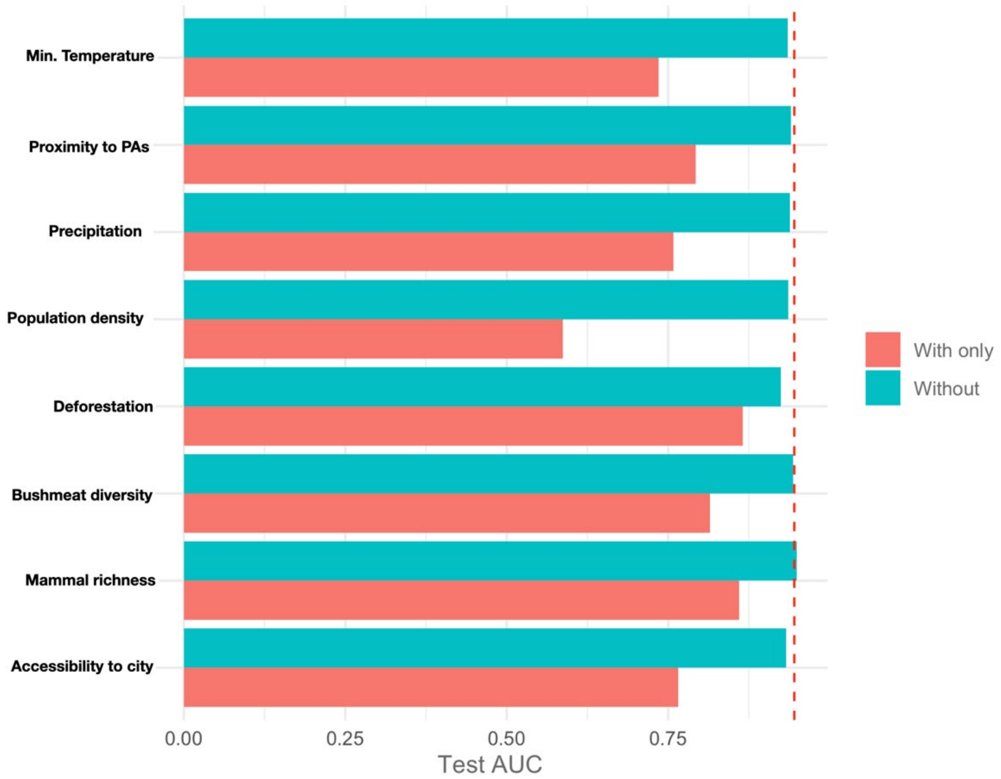

Appendix Figure 19. Jackknife test to test the variable contribution for the BRT model, permutation set to 10.

BRT: ROC

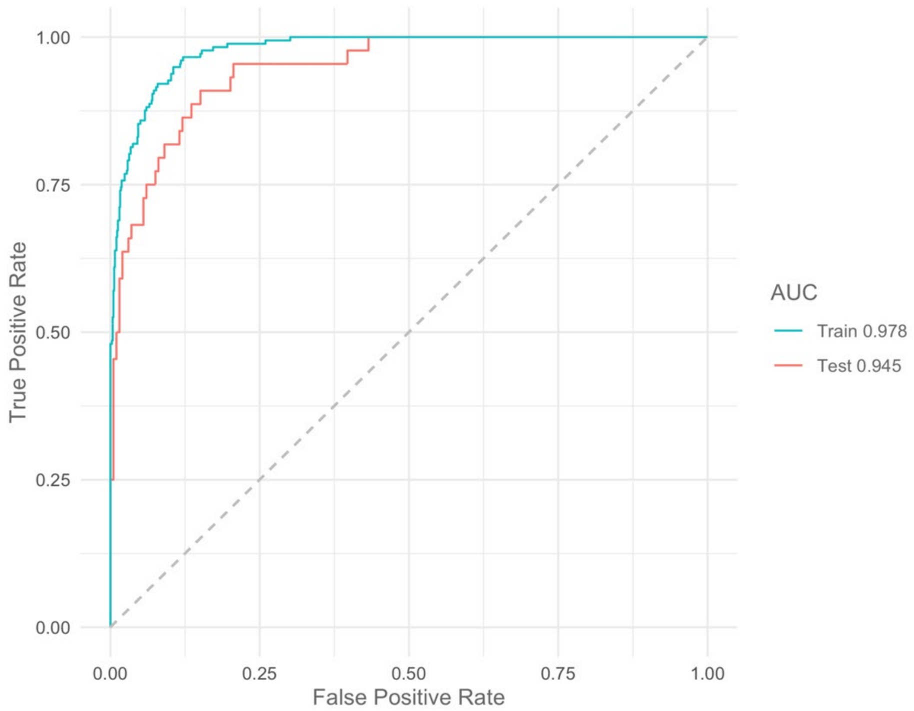

Appendix Figure 20. ROC curves of the BRT model.

BRT: Model prediction

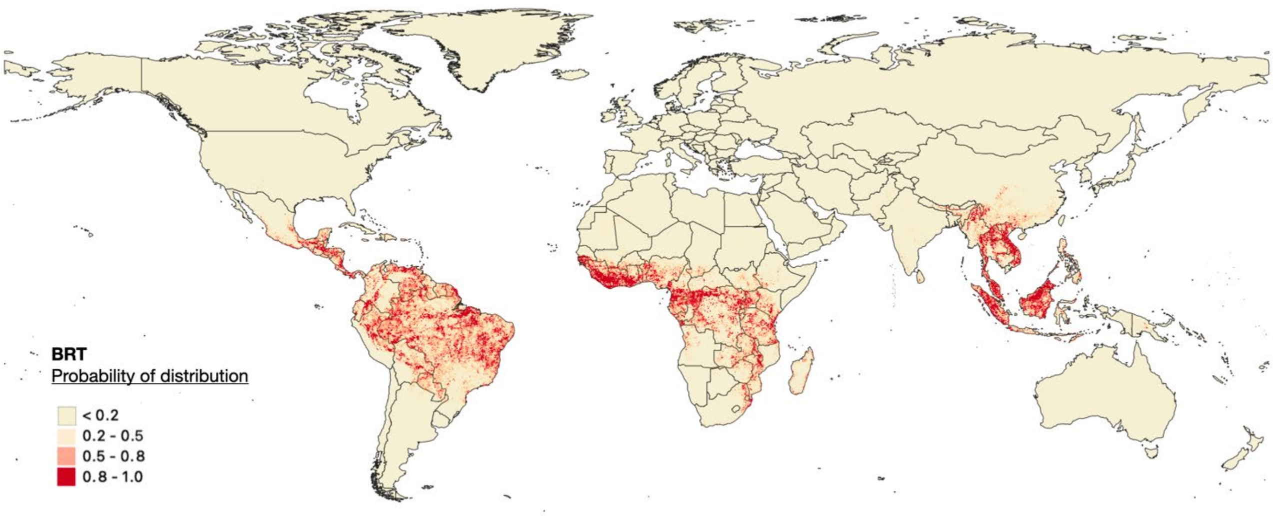

Appendix Figure 21. BRT model prediction of the distribution of bushmeat activities.

Maximum Entropy Model (Excluded Model)

We used R package dismo (*12*) for MaxENT modeling.

Appendix Table 13. MaxEnt model parameters

Metric	CV maxent	evnCV maxent	Final maxent
Area under the curve	0.8573559	0.7477496	0.9145729
True skill statistic	0.6452016	0.4771556	0.7255596

*CV, cross-validation; envCV, environmental cross-validation.

Although a TSS value between 0.4–0.5 is acceptable (*16*), we decided to impose more stringent threshold of 0.5 (*17*) across both the metrics. Moreover, the final prediction of MaxENT model led to non-convergence of the chains in the ensemble.

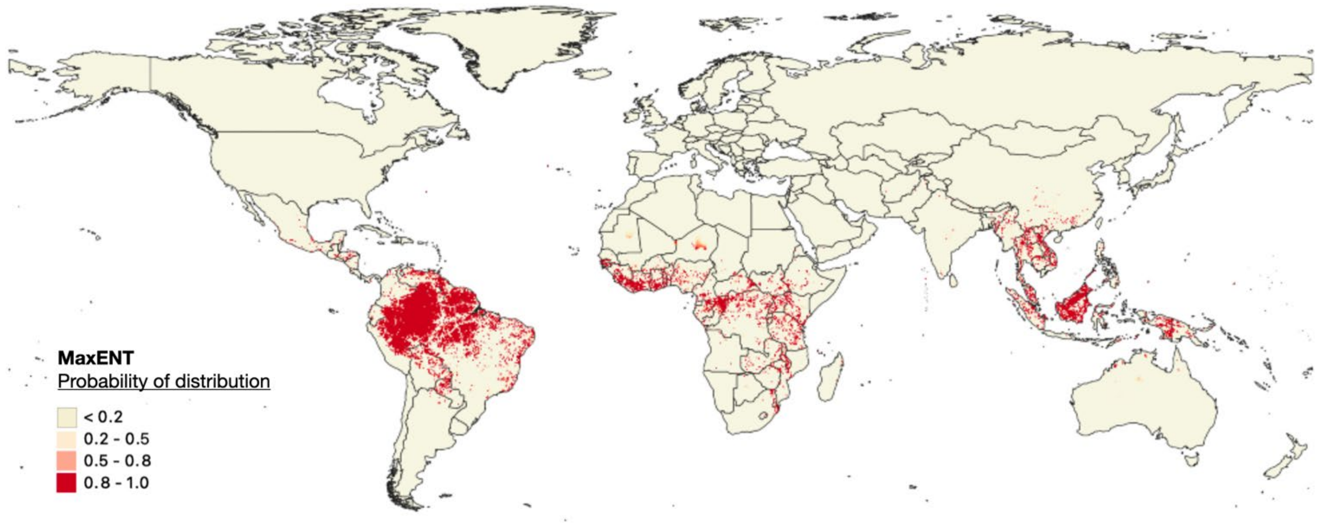

Appendix Figure 22. MaxENT model prediction excluded from the ensemble.

Bayesian Additive Regression Trees (BART) Model

We used R package 'embarcadero' (*18*) to fit the BART model.

Appendix Table 14. Bayesian additive regression tree (BART) model parameter metrics

Metric	BART
Area under the curve	0.9518754
True skill statistic	0.7750186

BART: Variable Importance

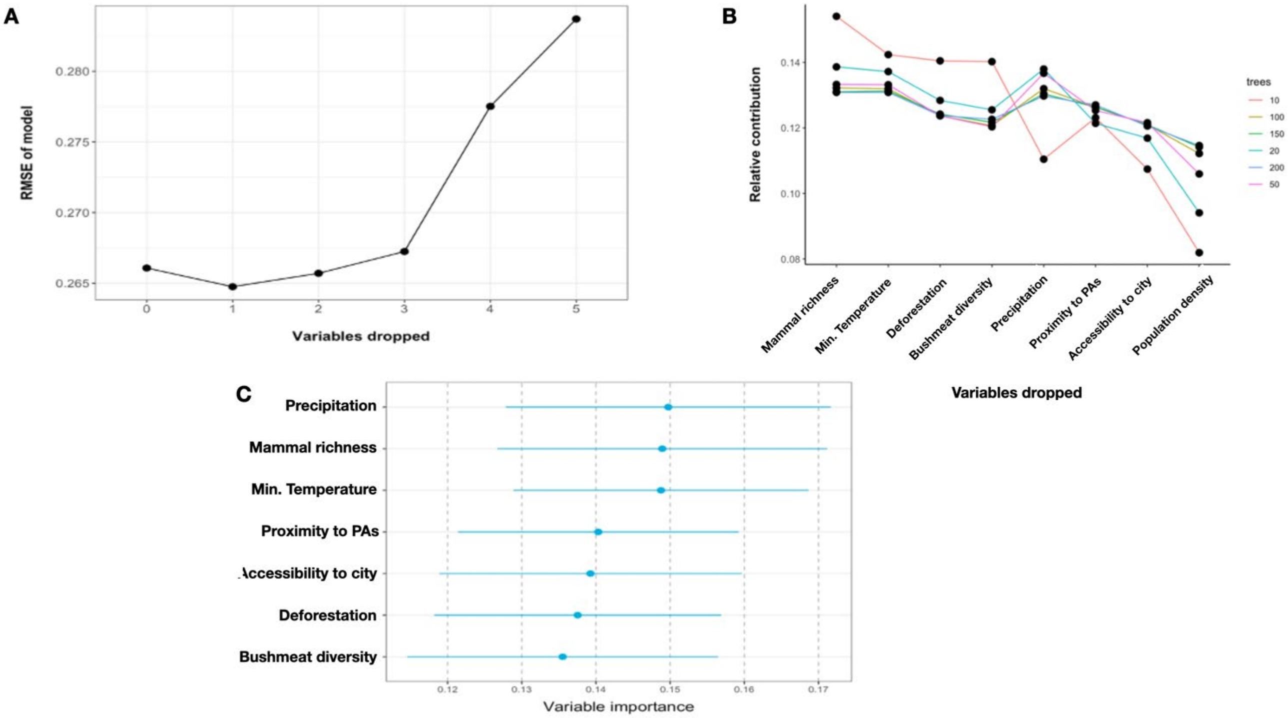

Appendix Figure 23. Plot illustrates the improvement of RMSE on dropping variables, B. Plot demonstrating the relative contribution calculated by dropping variables across the trees, C. Variable importance.

The above Figure 23, panel B shows that the prediction population density is to be dropped from the model as it fails to stay in the model when the number of trees drop to 10. For hyperparameter tuning of the BART model, we chose to use the default parameters a = 0.95 and b = 2 as recommended by a prior study (*19*).

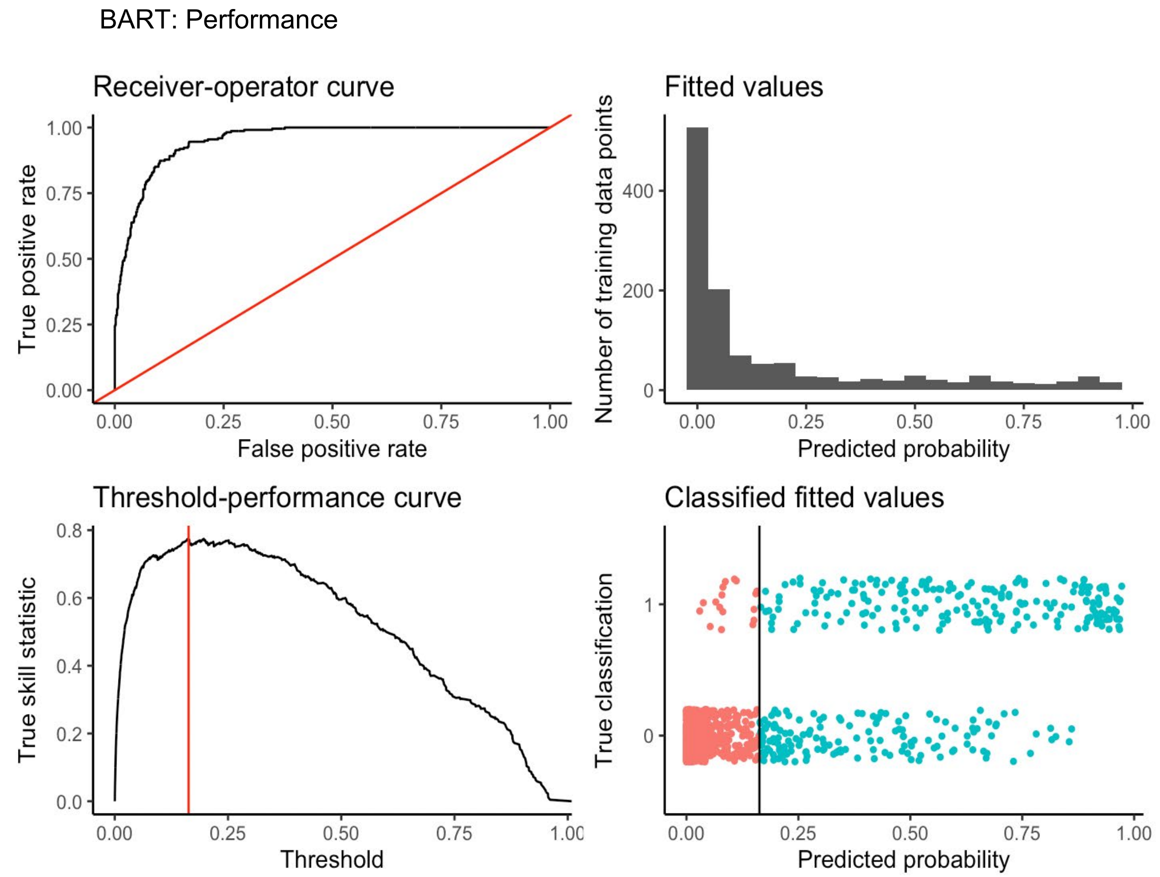

Appendix Figure 24. BART model performance diagnostic.

Area under the receiver-operator curve, AUC = 0.9518754.

Recommended threshold (maximizes true skill statistic), Cutoff = 0.162936; TSS = 0.7750186.

Resulting type I error rate: 0.05429864.

Resulting type II error rate: 0.1706827.

A high area under the ROC curve and clear visual split in the predicted probabilities assigned to the training presences and background points indicates that the model has done an adequate job (Appendix Figure 25).

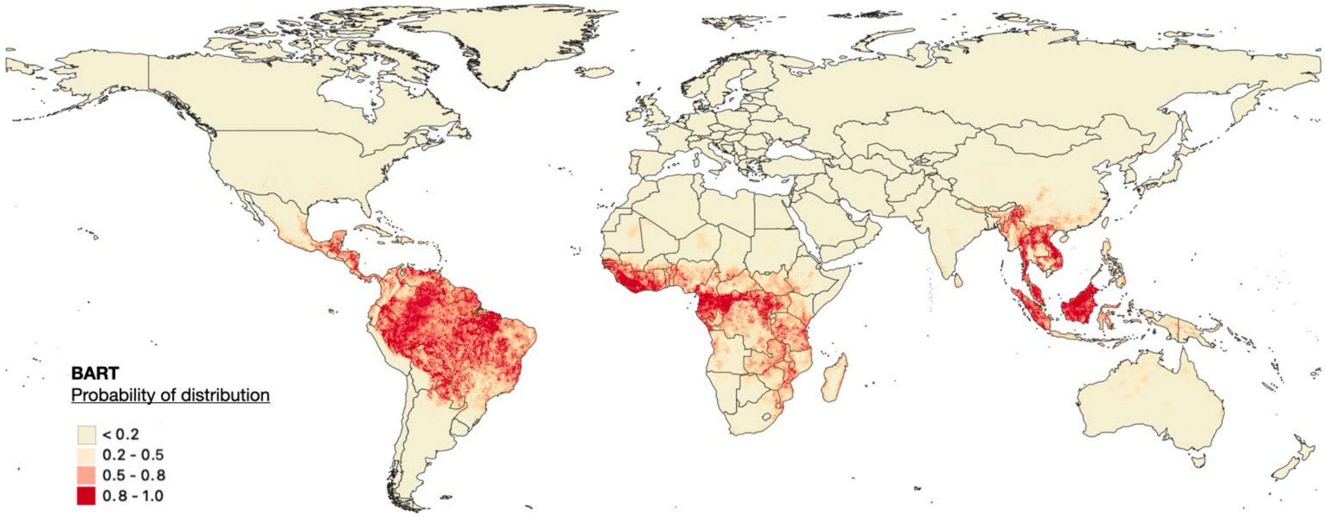

Appendix Figure 25. BART model prediction of the distribution of bushmeat activities.

Ensemble Model: Hierarchical Binomial Model with iCAR

In the final ensemble model for the distribution of bushmeat-related activities, the metacovariates (RF, BRT, and BART) were statistically significant and relevant to the final distribution (Appendix Tables 15, 16).

Appendix Table 15. Empirical mean and standard deviation for each variable

Variable	Mean	SD	Significance
β.(Intercept)	−4.505	0.2806	p<0.05
β.RandomForest	6.529	0.5348	p<0.05
β.BRT	1.970	0.4429	p<0.05
β.BART	−5.132	0.5789	p<0.05
Vrho	9.838	0.0737	
Deviance	235.874	16.1429	

Appendix Table 16. Quantiles for each variable

	Quantiles, %				
Variable	2.5	25	50	75	97.5
β.(Intercept)	−5.021	−4.701	−4.509	−4.305	−4.000
β.RandomForest	5.587	6.155	6.514	6.878	7.652
β.BRT	1.142	1.651	1.975	2.290	2.789
β.BART	−6.384	−5.506	−5.069	−4.730	−4.113
Vrho	9.723	9.779	9.831	9.887	9.993
Deviance	204.315	225.112	235.539	247.019	268.178

Model Convergence

Appendix Table 17. Gelman-Rubin's convergence metric for models

Parameter	Point estimate	Upper CI
β. (Intercept)	1.06	1.24
β.RandomForest	1.10	1.38
β.BRT	1.05	1.21
β.BART	1.01	1.01
Vrho	1.31	1.81
Deviance	1.01	1.04
Multivariate psrf	1.14	

The MCMC trace and density plots for binomial model in a hierarchical Bayesian framework with spatial autocorrelation (rho) with 4,000 iterations and 2 chains (2,000 per chain). We used non-informative priors with a large variance except for the spatial random effects, for which a uniform (min = 0, max = 10) weak informative prior was used for the parameter inference (Appendix Figure 26). Except for rho, the chains of the other parameters show convergence with regular density plots. While spatial autocorrelation parameter shows some areas of non-convergence with a slight irregularity in the density distribution, which is expected in the autocorrelation parameter. This is confirmed with the Gelman-Rubin's convergence metric (Appendix Table 17). Overall, the Gelman-Rubin's convergence diagnostic is 1.1 for the key parameters, thereby confirming convergence and the validity of the model.

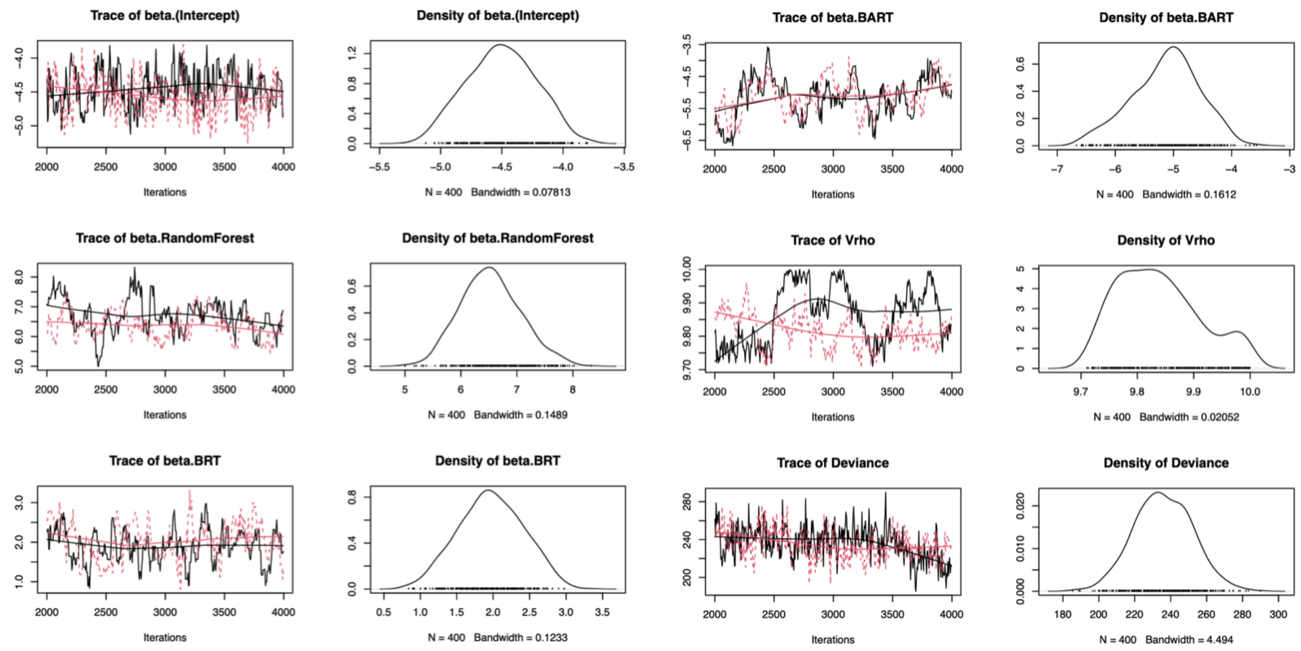

Appendix Figure 26. MCMC trace and density plots hierarchical binomial model with spatial autocorrelation.

Non-convergence on using the default rho prior (1/Gamma) had a multivariate potential scale reduction factor of 13.5 (Appendix Figure 27).

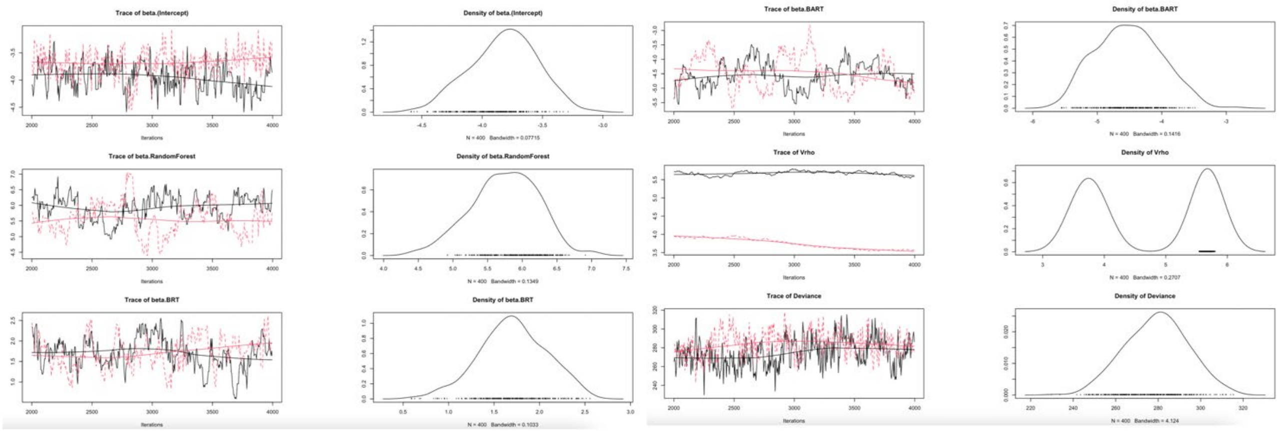

Appendix Figure 27. MCMC trace and density plots hierarchical binomial model with a default value for spatial autocorrelation.

Spatial Autocorrelation

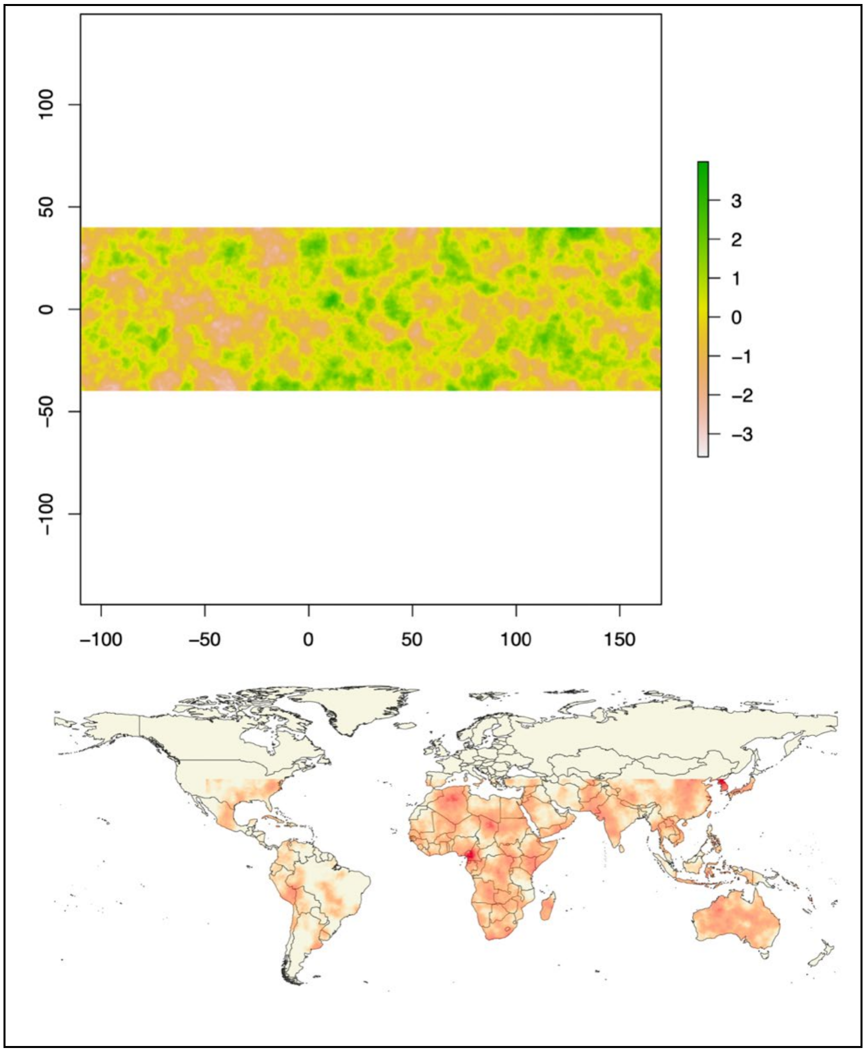

Appendix Figure 28. Spatial autocorrelation of the ensemble model.

The intrinsic conditional autoregressive model (iCAR) was calculated using:

p_i = Normal (u_i, V_p/n_i)

Where u_i = mean of p_i in the neighborhood of cell i; V_p = variance of the spatial random effects; n_i = number of neighbors for cell i adapted from a previous study (*20*). The iCAR used a spatial structure of the eight nearest neighboring pixels (Queen approach) to account for spatial correlation (Appendix Figure 28).

Distribution Threshold, Credible Intervals, and TSS

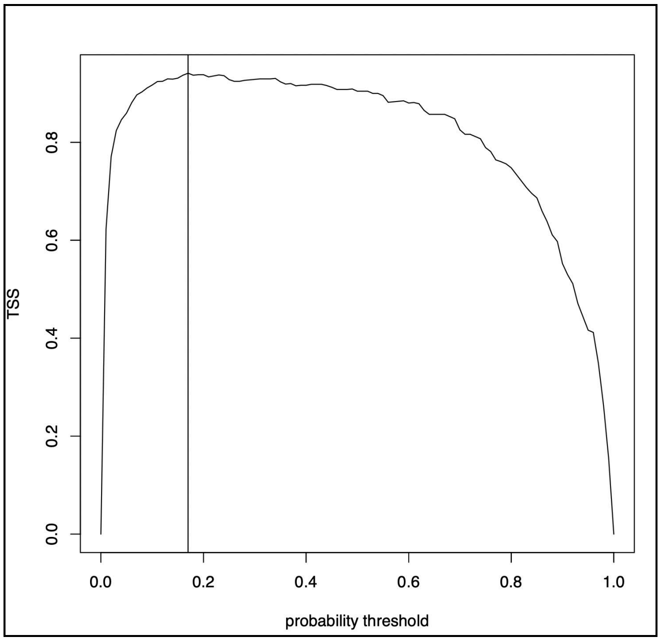

Appendix Figure 29. Plot of the True Skill Statistic against probability threshold identifying p = 0.17 as the probability threshold for a maximum TSS of 0.94.

We obtained a probability threshold of 0.17 (Appendix Figure 29) and a maximal TSS of 0.94. This is a relatively high TSS value, indicating a good correspondence between our predicted distribution area and observed presence and background sites. The high-risk area of bushmeat-related activities, defined as the 5×5-km cells with a presence probability value of 0.80 or greater, was 859,765.3 km^2 (Appendix Figures 30, 31)

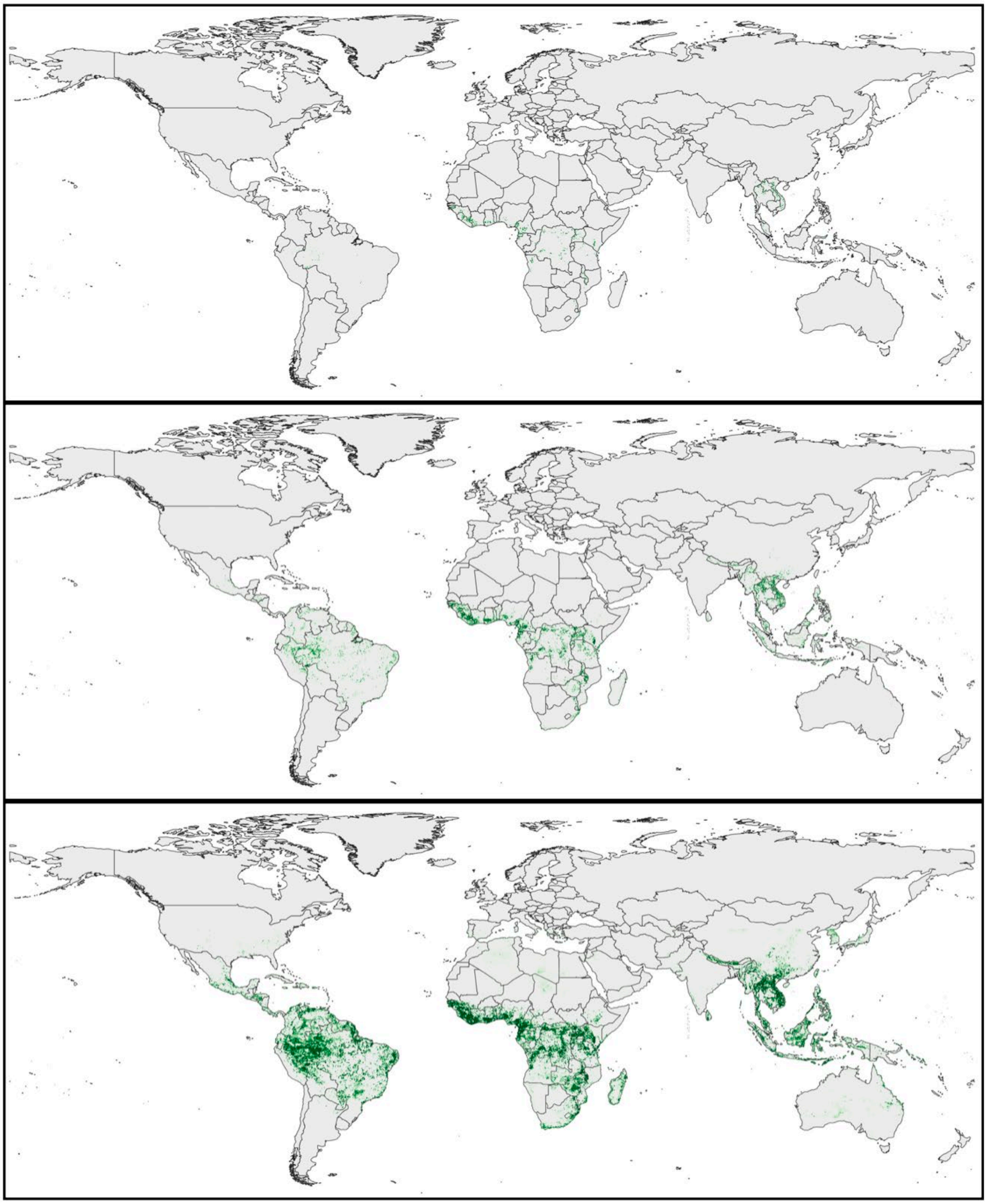

Appendix Figure 30. Plot of the lower bound (2.5%, top), mean (50%, center), and upper bound (97.5%, bottom) probability of distribution of bushmeat activities.

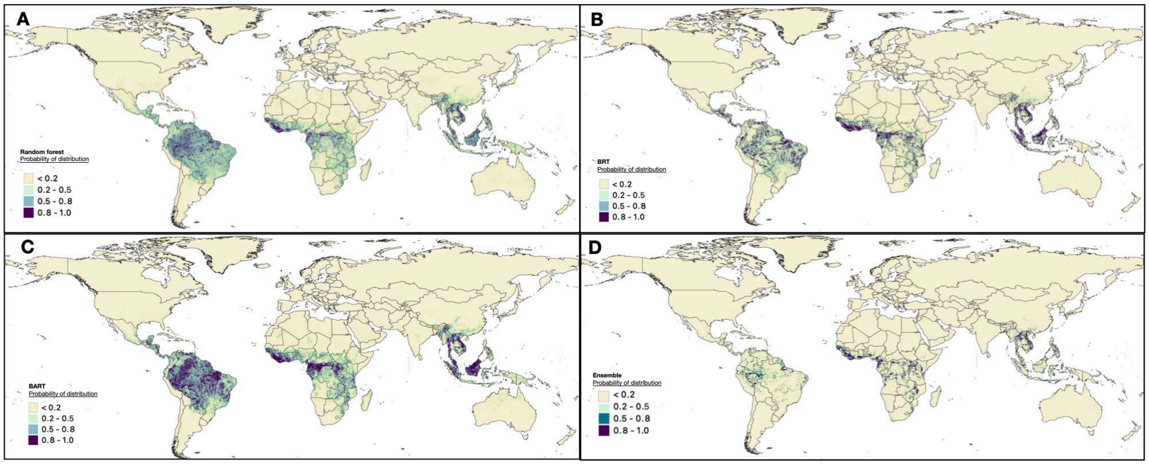

Appendix Figure 31. Overview of the model predictions demonstrating the distribution of bushmeat activities in a visual-friendly palette. A) Random Forest model; B) BRT model; C) BART mode; D) ensemble of the other models using hierarchical binomial regression.

Correlation between Mean Probability and Uncertainty of Ensemble

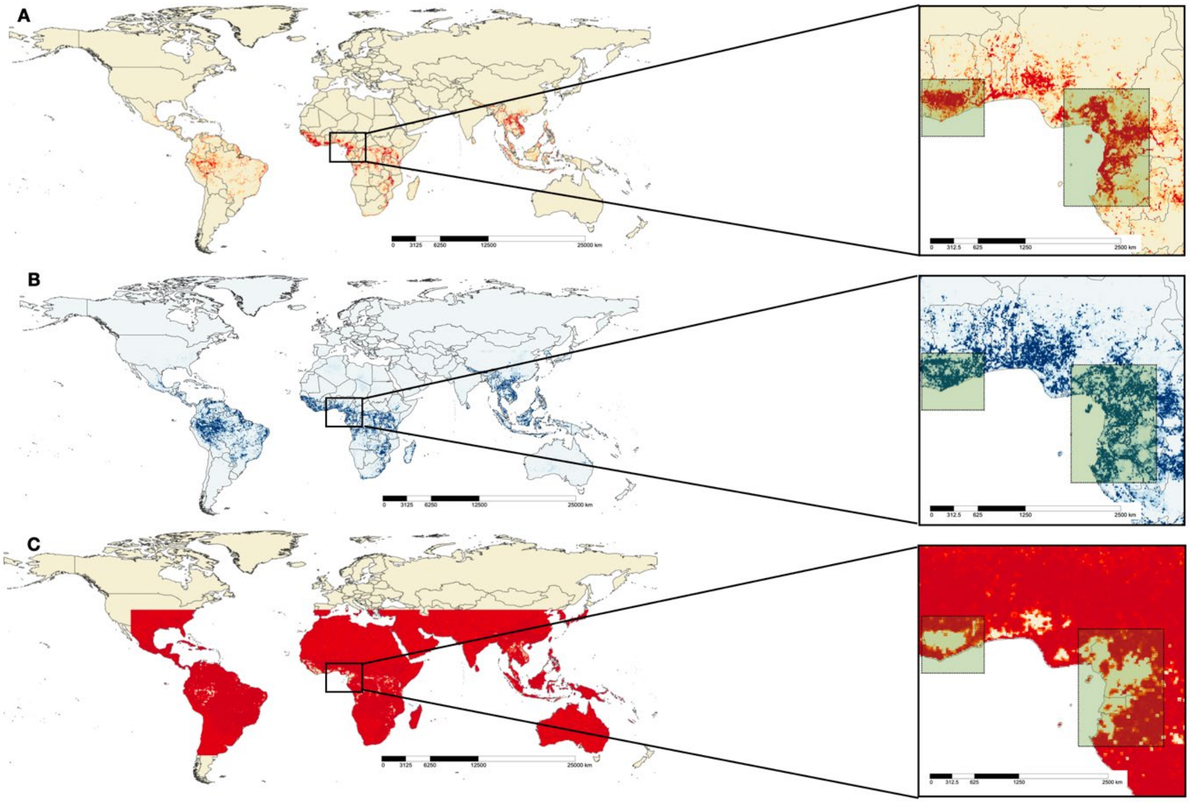

Appendix Figure 32. Correlation between mean probability and uncertainty of ensemble. A) Mean probability of distribution of bushmeat activities; B) uncertainty in bushmeat activities distribution; C) correlation between the mean probability and uncertainty.

Pearson's correlation was performed between the mean probability and uncertainty. We observed a negative correlation between the high mean probability pixels and uncertainty pixels, confirming that there is no correlation between the two rasters (Appendix Figure 32).

Areas Associated with Bushmeat Activities

Appendix Table 18. Areas and proportions of total area associated with bushmeat activities (BA)

Continent	Area associated with BA, km^2	% Total area with BA
Africa	55,1450.2	64.1
Central Africa	216,863.4	25.2
West Africa	188,945.2	22.0
East Africa	132,849.4	15.5
South Africa	12,792.3	1.5
Asia	214,961.9	24.9
South-East Asia	205,367.7	23.9
East Asia (China)	6,569.0	0.8
South Asia	2,074.4	0.2
Americas	93,353.2	11
South America	95,423.4	10.7
Central America	2,506.6	0.3
Total	859,765.3	100

Our results demonstrate that the area of Central Africa associated with bushmeat activities equals that the Asian continent and exceeds that of the Americas by over 2-folds (Appendix Table 18). Vietnam and Lao PDR contribute to half the area at risk (48.7%) in Asia. While Brazil and Peru contribute to 89.9% of the bushmeat risk in the Americas.

Necessity for Additional Surveillance

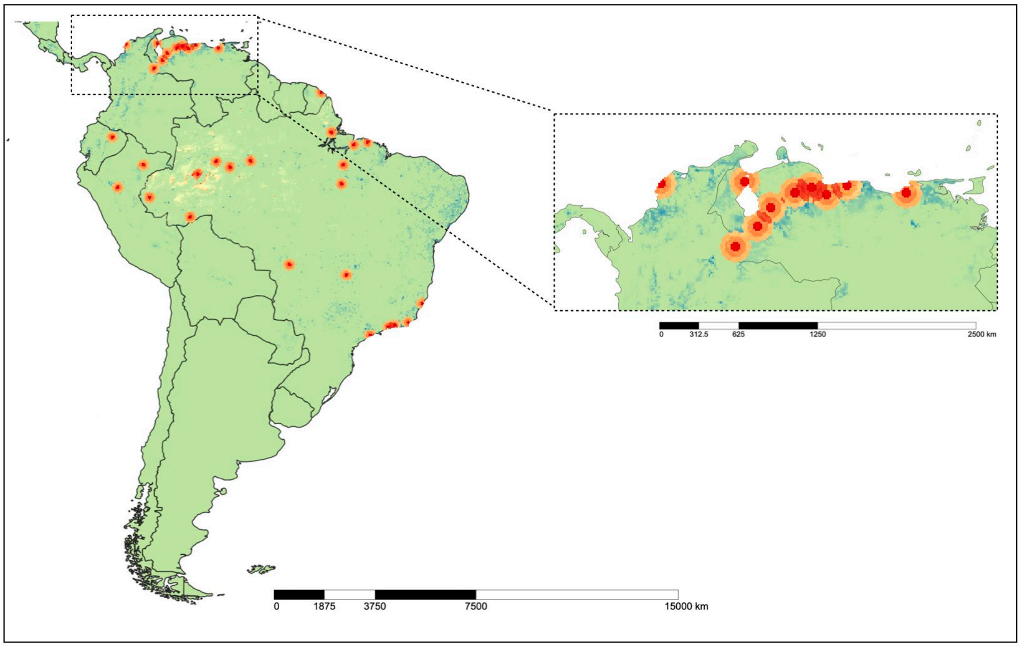

Appendix Figure 33. Necessity for additional surveillance map, product of the uncertainty, population density, and mammal richness, with survey locations of 50km radius.

The "necessity for additional surveillance" (NS) map (Appendix Figure 33), a product of the uncertainty, population density, and mammal richness. We predicted urban locations in the study area such as to minimize the mean NS_i across all the pixels in the map. The coordinates of the highest NS_i value (Appendix Table 19), was located on the NS map and the NS was reduced by a sequence of 25% in a 50-km radius around the site. A reduction of 75% is represented by the yellow, 50% by the orange, and 25% by the red concentric circles. The largest number of survey locations were found in Africa (52/100 surveys), followed by Americas (36/100 surveys), and Asia (12/100 surveys).

Appendix Table 19. List of locations that would benefit from future surveillance efforts

Latitude	Longitude	City	Country	Region	Remarks
−9.541667	16.375	Malanje	Angola	Central Africa	Bushmeat sales (*21*)
−12.375	16.95833	Kuito	Angola	Central Africa	
−11.79167	19.875	Luena	Angola	Central Africa	
−1.375	−48.375	Ananindeua	Brazil	South America	
−16.79167	−49.29167	Aparecda de Goiania	Brazil	South America	Roadkill vertebrate sale (*22*)
−5.125	−42.79167	Teressina	Brazil	South America	
−22.95833	−43.29167	Rio de Janeiro	Brazil	South America	
−15.625	−56.125	Cuiaba	Brazil	South America	Logging road Santarém–Cuiabá corridor (*23*)
−12.875	−38.375	Salvador	Brazil	South America	
−9.541667	−35.79167	Maceio	Brazil	South America	
−9.958333	−67.79167	Rio Branco	Brazil	South America	Commercial hunting (*24*)
−20.29167	−40.375	Cariacica	Brazil	South America	
−15.875	−48.125	Samambaia	Brazil	South America	
−20.79167	−49.375	Sao Jose do rio preto	Brazil	South America	
−5.541667	−47.45833	Imperatriz	Brazil	South America	
−8.791667	−63.875	Porto Velho	Brazil	South America	Commercial hunting (*24*)
−3.791667	−38.625	Fortaleza	Brazil	South America	Bushmeat trade (*25*)
−10.875	−37.04167	Aracaju	Brazil	South America	Bushmeat trade (*25*)
−23.95833	−46.45833	Sao Vicente	Brazil	South America	
−8.291667	−35.95833	Caruaru	Brazil	South America	
3.875	11.54167	Yaounde	Cameroon	Central Africa	Multiple reports of bushmeat trade
4.041667	9.708333	Douala	Cameroon	Central Africa	Large markets (*26*)
4.375	18.54167	Bangui	CAR	Central Africa	Multiple reports of bushmeat trade
27.70833	106.9583	Zunyi	China	Eastern Asia	
26.625	106.7083	Guiyang	China	Eastern Asia	
30.95833	103.625	Dujiangyan City	China	Eastern Asia	
10.375	−75.45833	Bolivar	Colombia	South America	Bushmeat trade (*27*)
4.625	−74.04167	Bogota	Colombia	South America	Bushmeat trade (*27*)
10.45833	−73.29167	Cesar	Colombia	South America	Bushmeat restaurants (*28*)
9.291667	−75.375	Sincelejo	Colombia	South America	
7.875	−72.45833	Villa Del Rosario	Colombia	South America	
3.458333	−76.45833	Palmira	Colombia	South America	
2.958333	−75.29167	Neiva	Colombia	South America	
7.041667	−73.125	Floridablanca	Colombia	South America	
5.458333	−4.041667	Abidjan	Cote d'Ivorie	Western Africa	Multiple reports of bushmeat trade
6.875	−6.458333	Daloa	Cote d'Ivorie	Western Africa	Primate sales (*29*)
6.791667	−5.291667	Yamoussoukro	Cote d'Ivorie	Western Africa	
6.125	−5.958333	Gagnoa	Cote d'Ivorie	Western Africa	
0.4583333	29.45833	Beni	DRC	Central Africa	Epicenter of 2018–20 Ebola outbreak
1.958333	30.04167	Djugu	DRC	Central Africa	
0.5416667	25.20833	Kisangani	DRC	Central Africa	Large market (*30*)
−5.875	22.375	Kananga	DRC	Central Africa	
2.291667	30.95833	Mahagi	DRC	Central Africa	
−4.458333	15.375	Kinshasa	DRC	Central Africa	Multiple reports of bushmeat trade
3.125	30.70833	Ariwara	DRC	Central Africa	

Latitude	Longitude	City	Country	Region	Remarks
0.04166667	18.29167	Mbandaka	DRC	Central Africa	Epicenter of multiple Ebola outbreaks including the 2022
−7.291667	27.375	Manono	DRC	Central Africa	
−5.041667	18.79167	Kikwit	DRC	Central Africa	Epicenter of the 1995 Ebola outbreak
−6.458333	20.79167	Tshikapa City	DRC	Central Africa	
−1.708333	29.04167	Kalehe	DRC	Central Africa	
−3.375	29.125	Uvira	DRC	Central Africa	
3.291667	19.79167	Gemena City	DRC	Central Africa	
2.791667	27.625	Isiro	DRC	Central Africa	Epicenter of the 2012 Ebola outbreak
−2.125	−79.95833	Guayaquil	Ecuador	South America	
−1.041667	−80.45833	Portoviejo	Ecuador	South America	
6.625	−1.625	Kumasi	Ghana	Western Africa	Atwemonom Market, Kumasi the largest bushmeat market in Ghana
5.541667	−0.458333	Kasoa	Ghana	Western Africa	
4.958333	−1.791667	Takoradi	Ghana	Western Africa	Bushmeat trade (*31*)
6.625	0.4583333	Ho	Ghana	Western Africa	
15.45833	−87.95833	San Pedro Sula	Honduras	Central America	
22.375	114.2083	Sha Tin	Hong Kong	Eastern Asia	
26.34567	89.29167	Assam	India	South Asia	Pangolin trade (*32*)
−5.375	105.2917	Lampung	Indonesia	South-Eastern Asia	
−2.958333	104.7917	Palembang	Indonesia	South-Eastern Asia	
1.375	99.29167	Padang Sidempuan	Indonesia	South-Eastern Asia	
−0.4583333	117.125	Samarinda	Indonesia	South-Eastern Asia	
6.291667	−10.70833	Paynesille	Liberia	Western Africa	Multiple reports of bushmeat trade
3.125	101.7917	Selangor	Malaysia	South-Eastern Asia	
4.625	101.125	Perak	Malaysia	South-Eastern Asia	
−19.125	33.45833	Chimoio	Mozambique	Eastern Africa	
−16.125	35.79167	Milange	Mozambique	Eastern Africa	
12.125	−86.29167	Managua	Nicaragua	Central America	
6.458333	3.291667	Lagos	Nigeria	Western Africa	Multiple reports of bushmeat trade
7.375	3.958333	Ibadan	Nigeria	Western Africa	Reports of mpox and bushmeat
8.458333	4.625	Erin	Nigeria	Western Africa	
7.625	5.208333	Ado Ekiti	Nigeria	Western Africa	Reports of mpox and bushmeat
5.125	7.375	Aba	Nigeria	Western Africa	
9.041667	7.458333	Abuja	Nigeria	Western Africa	Reports of mpox and bushmeat
5.041667	8.375	Calabar	Nigeria	Western Africa	
−8.375	−74.54167	Pucallpa	Peru	South America	Primate trade
−6.458333	−76.375	Tarapoto	Peru	South America	Commercial trade (*33*)
−2.541667	28.875	Rusizi	Rwanda	Western Africa	
8.458333	−13.20833	Freetown	Sierra Leone	Western Africa	
7.958333	−11.70833	Bo	Sierra Leone	Western Africa	Multiple reports of bushmeat trade
3.625	32.04167	Nimule	South Sudan	Central Africa	
4.875	31.54167	Juba	South Sudan	Central Africa	
−6.791667	39.20833	Dar es Salaam	Tanzania	Eastern Africa	Multiple reports of bushmeat trade
−2.541667	32.95833	Mwanza	Tanzania	Eastern Africa	
−9.291667	32.79167	Chapwas	Tanzania	Eastern Africa	
18.79167	99.04167	Chiang Mai	Thailand	South-Eastern Asia	
6.208333	1.125	Lome	Togo	Western Africa	
0.2083333	32.54167	Kajjansi	Uganda	Eastern Africa	
0.625	33.45833	Kigulu	Uganda	Eastern Africa	
10.04167	−69.375	Lara	Venezuela	South America	
10.625	−71.70833	Zulia	Venezuela	South America	
10.45833	−66.54167	Miranda	Venezuela	South America	
10.125	−67.95833	Valencia	Venezuela	South America	
9.708333	−63.20833	Monagas	Venezuela	South America	
−19.45833	29.79167	Gweru	Zimbabwe	Eastern Africa	
−19.95833	31.45833	Glencova	Zimbabwe	Eastern Africa	

*CAR, Central African Republic; DRC, Democratic Republic of Congo

Post-Hoc Validation

Model 1: MaxENT Ebola (*34*)

Occurrence data: Data available from the article.

Predictor variables: Worldclim, selected predictors mentioned in the article.

R code: Maxent version 3.4.4 used, code not needed.

Methods: Maxent model with 25% test data 1,000 replicates with maximum number of background points = 10,000, regularization multiplier 1, convergence threshold = 0.00001.

Model reproduced: Yes (Appendix Figure 34),

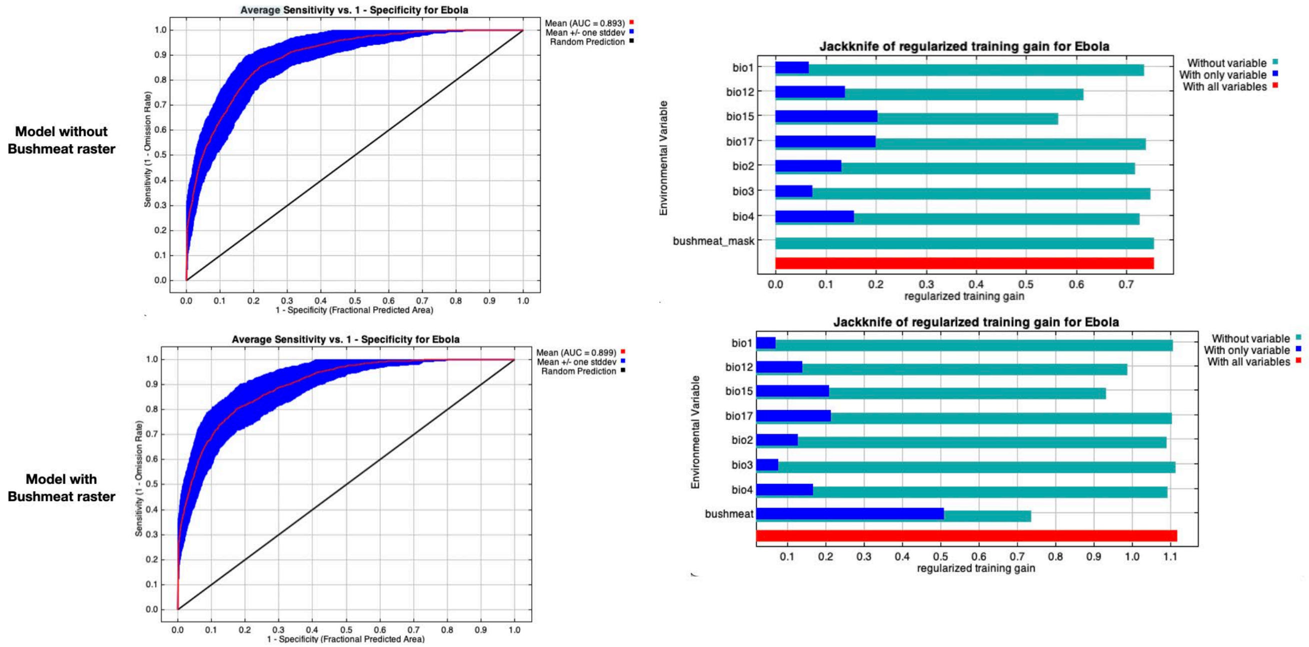

Appendix Figure 34. Ebola Model (Nyakarahuka et al. 2017) comparison between model without and with bushmeat activities raster.

Model 2: BRT Ebola (*35*)

Occurrence data: Data available from the article.

Predictor variables: bat distribution obtained from the author but unable to reproduce the temperature predictors (land surface temperature) to extensive cloud cover.

R code: Code available.

Methods: BRT model,The Ebola virus occurrence dataset was supplemented with a background record dataset generated by randomly sampling 10,000. Fitted 500 submodels to bootstraps of this dataset. Monte Carlo procedure enabled the model to efficiently integrate over the environmental uncertainty associated with imprecise geographic data. A bootstrap sample was then taken from each of these datasets and used to train the BRT model.

Model reproduced: Yes

The model reproduced with a randomly permutated BA values as one of the covariates had a mean AUC of 0.880 (Appendix Table 20).

Appendix Table 20. Reproducibility of BRT Ebola model with a randomly permutated bushmeat activity values

Relative influence	Mean	2.5%	97.5%
EVI mean	53.1	38.6	63.4
tmax_range	16.0	7.7	31.9
PET	7.4	4.9	10.3
Altitude	5.8	4.4	7.9
tmin_r	5.1	4.1	6.5
tmin_m	4.1	2.7	5.3
Permutated BA	3.3	2.1	4.8
Bat distribution	2.5	1.5	3.8
tmax_mean	1.8	0.8	2.9
EVI range	0.9	0.2	2.1

The model reproduced with the bushmeat variables as on of the predictor covariates had a mean AUC of 0.887 (Appendix Table 21).

Appendix Table 21. Reproducibility of BRT Ebola model with bushmeat variables

Relative influence	Mean	2.50%	97.50%
EVI mean	50.0	37.8	61.8
Bushmeat	17.1	7.3	30.8
tmax_range	7.3	5.0	9.2
PET	6.3	4.2	8.3
tmax_mean	5.5	3.8	7.1
tmin_range	4.5	3.2	5.5
Bat distribution	3.7	2.8	4.8
tmin_mean	2.8	1.4	3.8
Altitude	1.9	0.8	3.0
EVI range	0.9	0.4	1.8

Model 3: Excluded MaxENT mpox (*36*)

Occurrence data: not available. mpox case located extracted from WHO and CDC reports (Data available on GitHub)

Predictor variables: Worldclim, selected predictors mentioned in the article.

R code: Maxent version 3.4.4 used, code not needed.

Methods: Maxent model with 25% test data with maximum number of background points = 10,000, regularization multiplier 1, convergence threshold = 0.00001.

Model reproduced: No, thus excluded from the results (Appendix Figure 35).

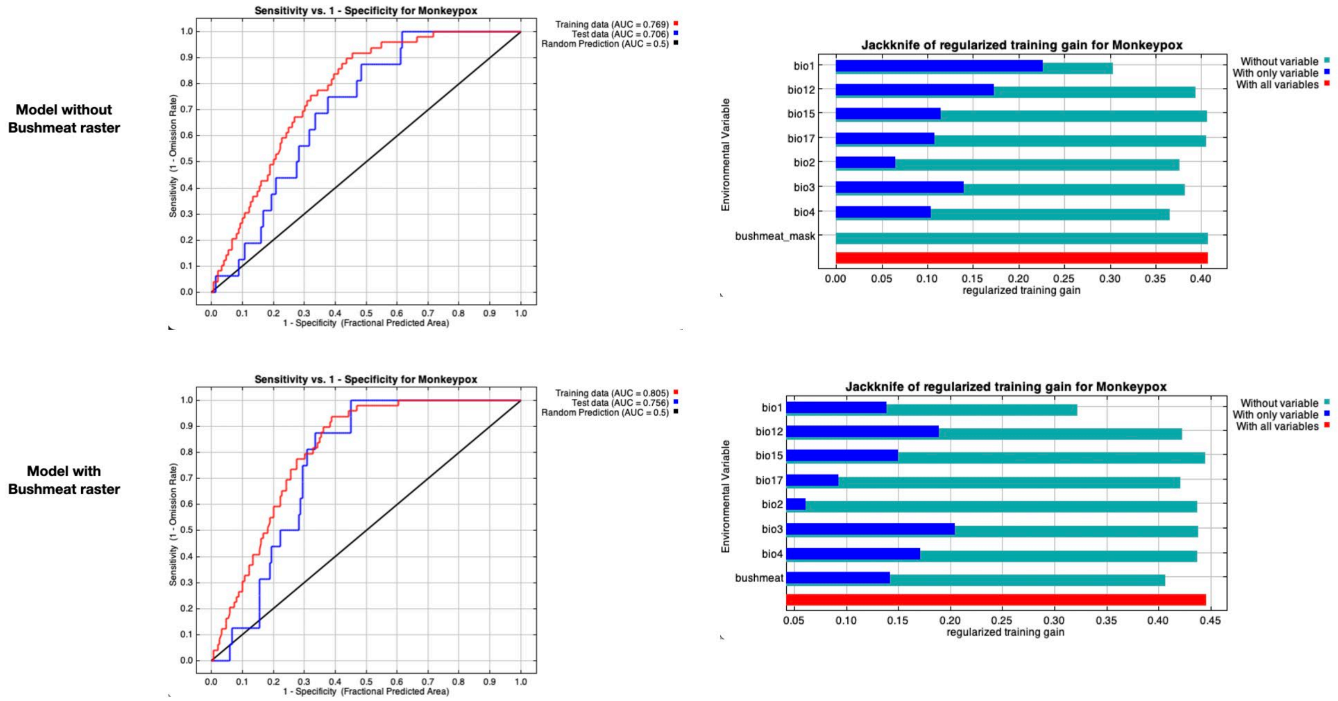

Appendix Figure 35. Mpox Model (*36*) comparison between model without and with bushmeat activities raster.

References

1. Milner-Gulland EJ, Bennett EL. Wild meat: the bigger picture. Trends Ecol Evol. 2003;18:351–7. https://doi.org/10.1016/S0169-5347(03)00123-X

2. Hansen MC, Potapov PV, Moore R, Hancher M, Turubanova SA, Tyukavina A, et al. High-resolution global maps of 21st-century forest cover change. Science. 2013;342:850–3. PubMed https://doi.org/10.1126/science.1244693

3. International Union for Conservation of Nature; Center for International Earth Science Information Network Columbia University. Gridded species distribution: global mammal richness grids [cited 2022 May 10]. Palisades (NY): NASA Socioeconomic Data and Applications Center (SEDAC); 2015. https://doi.org/10.7927/H4N014G5

4. International Union for Conservation of Nature and Natural Resources. IUCN red list of threatened species [cited 2022 Mar 30]. https://www.iucnredlist.org/en

5. Nelson A, Weiss DJ, van Etten J, Cattaneo A, McMenomy TS, Koo J. A suite of global accessibility indicators. Sci Data. 2019;6:266. PubMed https://doi.org/10.1038/s41597-019-0265-5

6. International Union for Conservation of Nature; Center for International Earth Science Information Network Columbia University. Gridded population of the world, version 4 (GPWv4): population density, revision 11 [cited 2022 May 10]. Palisades (NY): NASA Socioeconomic Data and Applications Center (SEDAC); 2018. https://doi.org/10.7927/H49C6VHW

7. Kummu M, Taka M, Guillaume JHA. Gridded global datasets for Gross Domestic Product and Human Development Index over 1990–2015. Sci Data. 2018;5:180004. PubMed https://doi.org/10.1038/sdata.2018.4

8. Ripple WJ, Abernethy K, Betts MG, Chapron G, Dirzo R, Galetti M, et al. Bushmeat hunting and extinction risk to the world's mammals. R Soc Open Sci. 2016;3:160498. PubMed https://doi.org/10.1098/rsos.160498

9. Nasi R, Taber A, Van Vliet N. Empty forests, empty stomachs? Bushmeat and livelihoods in the Congo and Amazon Basins. Int Rev. 2011;13:355–68. https://doi.org/10.1505/146554811798293872

10. Fa JE, Wright JH, Funk SM, Márquez AL, Olivero J, Farfán MÁ, et al. Mapping the availability of bushmeat for consumption in Central African cities. Environ Res Lett. 2019;14:094002. https://doi.org/10.1088/1748-9326/ab36fa

11. Liaw A, Wiener M. Classification and regression by randomForest. R News. 2002;2(3):18–22.

12. Hijmans RJ, Leathwick J, Phillips S, Elith J. Dismo: species distribution modeling [cited 2022 May 10]. https://rspatial.org/raster/sdm

13. Breiman L. Random forests. Mach Learn. 2001;45:5–32. https://doi.org/10.1023/A:1010933404324

14. Greenwell B, Boehmke B, Cunningham J, Developers G. gbm: Generalized boosted regression models [cited 2022 May 10]. https://cran.r-project.org/web/packages/gbm/index.html

15. Elith J, Leathwick JR, Hastie T. A working guide to boosted regression trees. J Anim Ecol. 2008;77:802–13. PubMed https://doi.org/10.1111/j.1365-2656.2008.01390.x

16. Zhang L, Liu S, Sun P, Wang T, Wang G, Zhang X, et al. Consensus forecasting of species distributions: the effects of niche model performance and niche properties. PLoS One. 2015;10:e0120056. PubMed https://doi.org/10.1371/journal.pone.0120056

17. Liu C, White M, Newell G. Measuring and comparing the accuracy of species distribution models with presence-absence data. Ecography. 2011;34:232–43. https://doi.org/10.1111/j.1600-0587.2010.06354.x

18. Carlson CJ. embarcadero: Species distribution modelling with Bayesian additive regression trees in R. Methods Ecol Evol. 2020;11:850–8. https://doi.org/10.1111/2041-210X.13389

19. Chipman HA, George EI, McCulloch RE. BART: Bayesian additive regression trees. Ann Appl Stat. 2010;4:266–98. https://doi.org/10.1214/09-AOAS285

20. Plumptre AJ, Nixon S, Kujirakwinja DK, Vieilledent G, Critchlow R, Williamson EA, et al. Catastrophic decline of world's largest Primate: 80% loss of Grauer's gorilla (*Gorilla beringei graueri*) population justifies critically endangered status. PLoS One. 2016;11:e0162697. PubMed https://doi.org/10.1371/journal.pone.0162697

21. Teutloff N, Meller P, Finckh M, Cabalo AS, Ramiro GJ, Neinhuis C, et al. Hunting techniques and their harvest as indicators of mammal diversity and threat in Northern Angola. Eur J Wildl Res. 2021;67:101. PubMed https://doi.org/10.1007/s10344-021-01541-y

22. Cunha H, Moreira F, Silva S. Roadkill of wild vertebrates along the GO-060 road between Goiânia and Iporá, Goiás State, Brazil. Acta Sci Biol Sci. 2010;32:257–63. https://doi.org/10.4025/actascibiolsci.v32i3.4752

23. Soares-Filho B, Alencar A, Nepstad D, Cerqueira G, Vera Diaz MC, Rivero S, et al. Simulating the response of land-cover changes to road paving and governance along a major Amazon highway: the Santarém–Cuiabá corridor. Glob Change Biol. 2004;10:745–64. https://doi.org/10.1111/j.1529-8817.2003.00769.x

24. Food and Agriculture Organization. Commercial hunting [cited 2022 Jun 9]. https://www.fao.org/3/t0750e/t0750e07.htm

25. Ferreira FS, Albuquerque U, Coutinho H, Almeida WO, Alves RR. The trade in medicinal animals in northeastern Brazil. Evid Based Complement Alternat Med. 2012;2012:126938. PubMed https://doi.org/10.1155/2012/126938

26. Fa JE, Seymour S, Dupain J, Amin R, Albrechtsen L, Macdonald D. Getting to grips with the magnitude of exploitation: Bushmeat in the Cross–Sanaga rivers region, Nigeria and Cameroon. Biol Conserv. 2006;129:497–510. https://doi.org/10.1016/j.biocon.2005.11.031

27. Gómez J, van Vliet N, Restrepo S, Daza E, Moreno J, Cruz-Antia D, et al. Use and trade of bushmeat in Colombia: Relevance to rural livelihoods. Center for International Forestry Research [cited 2022 Jan 20]. https://www.jstor.org/stable/resrep16220

27. Center for International Forestry Research. Bushmeat and livelihoods: a journey throughout the regions from Colombia [cited 2022 Jun 9]. https://www2.cifor.org/bushmeat/bushmeat-livelihoods-journey-throughout-regions-colombia

29. Covey R, McGraw WS. Monkeys in a West African bushmeat market: implications for Cercopithecid conservation in eastern Liberia. Trop Conserv Sci. 2014;7:115–25. https://doi.org/10.1177/194008291400700103

30. van Vliet N, Nebesse C, Gambalemoke S, Akaibe D, Nasi R. The bushmeat market in Kisangani, Democratic Republic of Congo: implications for conservation and food security. Oryx. 2012;46:196–203. https://doi.org/10.1017/S0030605311000202

31. Mendelson S, Cowlishaw G, Rowcliffe JM. Anatomy of a bushmeat commodity chain in Takoradi, Ghana. J Peasant Stud. 2003;31:73–100. https://doi.org/10.1080/0306615031000169341

32. D'Cruze N, Singh B, Mookerjee A, Harrington LA, Macdonald DW. A socio-economic survey of pangolin hunting in Assam, northeast India. Nat Conserv. 2018;30:83–105. https://doi.org/10.3897/natureconservation.30.27379

33. van Vliet N, Quiceno MP, Cruz D, de Aquino LJN, Yagüe B, Schor T, et al. Bushmeat networks link the forest to urban areas in the Trifrontier Region between Brazil, Colombia, and Peru. Ecol Soc. 2015;20:art21. https://doi.org/10.5751/ES-07782-200321

34. Nyakarahuka L, Ayebare S, Mosomtai G, Kankya C, Lutwama J, Mwiine FN, et al. Ecological niche modeling for filoviruses: a risk map for Ebola and Marburg virus disease outbreaks in Uganda. PLoS Curr. 2017;9:ecurrents.outbreaks.07992a87522e1f229c7cb023270a2af1. PubMed https://doi.org/10.1371/currents.outbreaks.07992a87522e1f229c7cb023270a2af1

35. Pigott DM, Golding N, Mylne A, Huang Z, Henry AJ, Weiss DJ, et al. Mapping the zoonotic niche of Ebola virus disease in Africa. eLife. 2014;3:e04395. PubMed https://doi.org/10.7554/eLife.04395

36. Fuller T, Thomassen HA, Mulembakani PM, Johnston SC, Lloyd-Smith JO, Kisalu NK, et al. Using remote sensing to map the risk of human monkeypox virus in the Congo Basin. EcoHealth. 2011;8:14–25. PubMed https://doi.org/10.1007/s10393-010-0355-5
